# The protease SPRTN and SUMOylation coordinate DNA-protein crosslink repair to prevent genome instability

**DOI:** 10.1016/j.celrep.2021.110080

**Published:** 2021-12-07

**Authors:** Annamaria Ruggiano, Bruno Vaz, Susan Kilgas, Marta Popović, Gonzalo Rodriguez-Berriguete, Abhay N. Singh, Geoff S. Higgins, Anne E. Kiltie, Kristijan Ramadan

**Affiliations:** 1 Medical Research Council (MRC) Oxford Institute for Radiation Oncology, Department of Oncology, University of Oxford, Roosevelt Drive, Oxford OX3 7DQ, UK; 2 Laboratory for Molecular Ecotoxicology, Division for Marine and Environmental Research, Ruđer Bošković Institute, Bijenička cesta 54, 10000 Zagreb, Croatia

**Keywords:** BRCA deficiency, DNA-protein crosslink repair, DNA replication, genome stability, formaldehyde toxicity, homologous recombination, synthetic lethality, SPRTN protease, SUMO, ubiquitin

## Abstract

DNA-protein crosslinks (DPCs) are a specific type of DNA lesion in which proteins are covalently attached to DNA. Unrepaired DPCs lead to genomic instability, cancer, neurodegeneration, and accelerated aging. DPC proteolysis was recently identified as a specialized pathway for DPC repair. The DNA-dependent protease SPRTN and the 26S proteasome emerged as two independent proteolytic systems. DPCs are also repaired by homologous recombination (HR), a canonical DNA repair pathway. While studying the cellular response to DPC formation, we identify ubiquitylation and SUMOylation as two major signaling events in DNA replication-coupled DPC repair. DPC ubiquitylation recruits SPRTN to repair sites, promoting DPC removal. DPC SUMOylation prevents DNA double-strand break formation, HR activation, and potentially deleterious genomic rearrangements. In this way, SUMOylation channels DPC repair toward SPRTN proteolysis, which is a safer pathway choice for DPC repair and prevention of genomic instability.

## Highlights

• SUMOylation and ubiquitylation enable DPC repair during DNA replication
• Resolution of ubiquitylated and SUMOylated DPCs requires the protease SPRTN
• Inactivation of SUMOylation after DPC formation activates homologous recombination
• DPC SUMOylation and SPRTN prevent recombination-dependent genomic instability

## Graphical abstract

**Figure:**
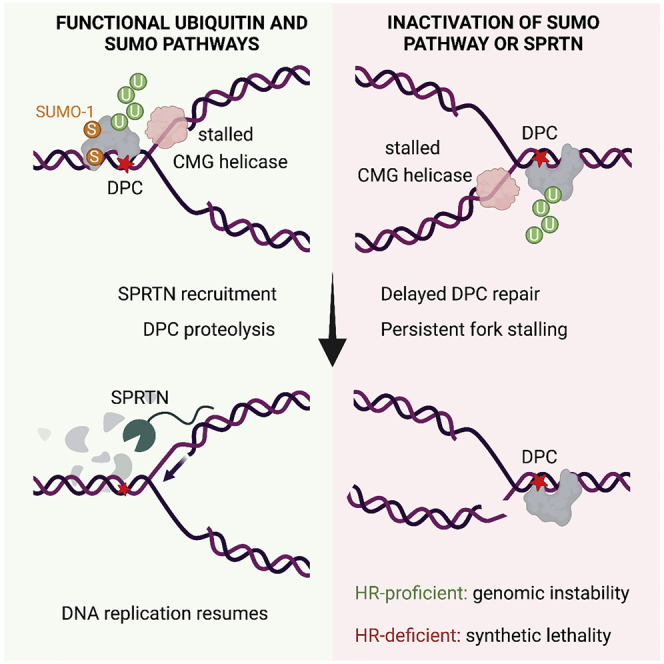

## Teaser

Ruggiano et al. show that ubiquitylation and SUMOylation of DNA-protein crosslinks is linked to repair by SPRTN during DNA replication. Blocking SUMOylation causes double-strand breaks and switches the repair pathway to homologous recombination, potentially causing chromosomal rearrangements and genomic instability. Inactivation of DPC repair causes synthetic lethality in recombination-defective cells.

## Introduction

DNA-protein crosslinks (DPCs) are ubiquitous and heterogeneous DNA lesions that arise from covalent binding of a protein to DNA following exposure to a chemical or physical crosslinking agent, e.g., formaldehyde (FA) or UV light (Ide et al., 2018; Kühbacher and Duxin, 2020; Vaz et al., 2017). FA is a cellular by-product of methanol metabolism, histone demethylation, and lipid peroxidation as well as an environmental pollutant. It is estimated that intracellular FA concentrations can reach 400 μM (Andersen et al., 2010), implying that threats posed by DPCs are ubiquitous. Furthermore, some of the most commonly used chemotherapeutics, namely the topoisomerases (Topo) 1 and 2 poisons camptothecin (CPT) and etoposide, respectively, cause abortive topoisomerase activity on DNA; this then causes a specific class of DPCs known as Topo-1 or Topo-2 cleavage complexes (Topo-1/2-ccs) (Ashour et al., 2015; Pommier and Marchand, 2011). Due to the stability of the crosslink and their bulkiness, DPCs constitute a barrier to all DNA transactions. If left unrepaired, DPCs lead to genomic instability and/or cell death as well as conditions including neurodegeneration, cancer, and premature aging in humans and mice (Gómez-Herreros et al., 2014; Lessel et al., 2014; Maskey et al., 2014, 2017). To cope with DPC-induced toxicity, cells employ two major repair pathways: (1) a proteolytic-dependent mechanism, where the proteinaceous component of the DPC is cleaved by specific proteases; and (2) a nucleolytic-dependent mechanism, where the nucleases involved in homologous recombination (HR) or nucleotide excision repair cleave off the DNA bearing a crosslinked protein (Aparicio et al., 2016; Hoa et al., 2016; Nakano et al., 2007, 2009). The former mechanism involves DNA-dependent metalloproteases, SPRTN in metazoans and Wss1 in yeast (Lopez-Mosqueda et al., 2016; Maskey et al., 2017; Mórocz et al., 2017; Stingele et al., 2014, 2016; Vaz et al., 2016), or the proteasome (Larsen et al., 2019; Sparks et al., 2019; Sun et al., 2020). In addition to SPRTN, several proteases, such as ACRC, also known as germ cell nuclear antigen (GCNA), FAM111A and FAM111B, and DDI1 and DDI2, have recently been discovered and linked to DPC proteolysis repair (reviewed in Ruggiano and Ramadan, 2021a). However, among them, *SPRTN* is the only essential gene in cells, indicating the crucial role of the protease SPRTN in DPC repair, embryogenesis, and cell survival.

While both proteolytic and nucleolytic pathways protect cells from DPC-induced toxicity, they come with downsides. HR can lead to aberrant genomic rearrangements and loss of heterozygosity (Liu et al., 2012), while proteolytic pathways can increase mutagenesis (Mórocz et al., 2017; Nakazato et al., 2018; Stingele et al., 2014). However, it is not known how DPC pathway choice between proteolysis and HR is coordinated.

Recently, post-translational modifications on DPCs by ubiquitin or small ubiquitin-like modifier (SUMO) molecules have emerged as two signals that govern proteolysis-dependent DPC repair: DPC ubiquitylation promotes proteolysis by the 26S proteasome, while DPC SUMOylation promotes ACRC recruitment to DPC lesions and their repair outside DNA replication (Borgermann et al., 2019; Larsen et al., 2019; Sparks et al., 2019; Sun et al., 2020). Considering that ACRC is expressed predominantly in germ and stem cells and not in human primary or cancer cell lines, it remains unclear why SUMOylation is important for DPC repair in proliferative somatic human cells.

Here, we report that both SUMOylation and transient ubiquitylation are required for SPRTN-dependent DPC repair during replication. Ubiquitylation is crucial for SPRTN’s binding to its substrates, localization to nuclear foci, and effective DPC repair. In parallel, SUMOylation suppresses HR-mediated recombinogenesis. Simultaneous inactivation of SPRTN-dependent proteolysis and the HR pathway leads to synthetic lethality after FA exposure, suggesting that SPRTN and HR act in parallel to prevent DPC-induced toxicity. We propose that SUMOylation channels DPC repair pathway choice toward SPRTN-dependent proteolysis to prevent recombinogenic events that could lead to genomic instability.

## Results

### DPCs are modified by SUMO and ubiquitin

**Figure 1:**
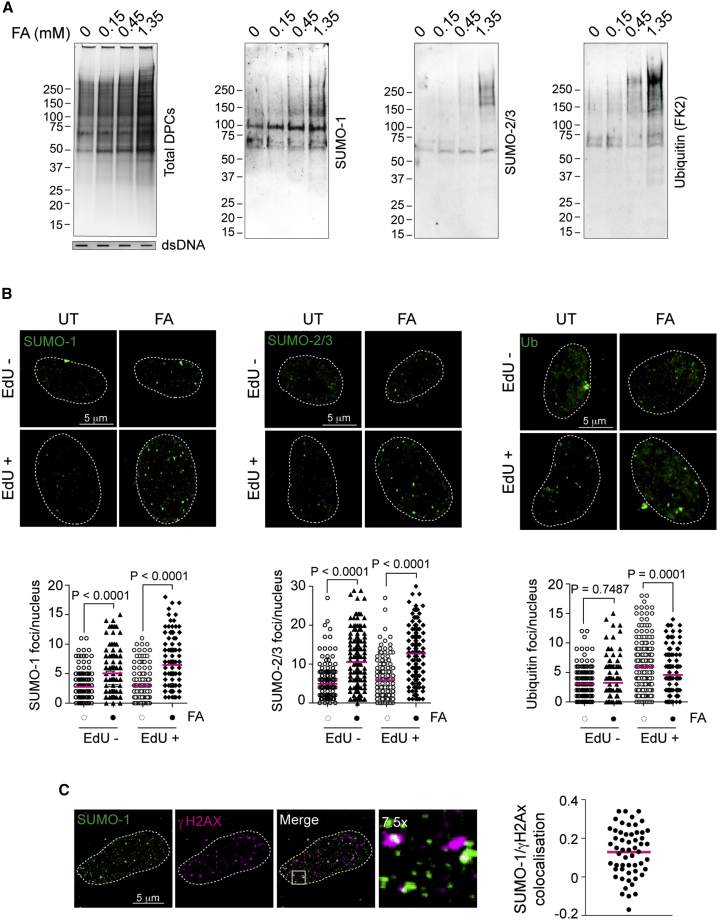
DPCs are modified by SUMO and ubiquitin (A) Formaldehyde (FA) treatment promotes ubiquitylation and SUMOylation on DPCs. HeLa cells were treated with increasing concentrations of FA for 10 min at 37°C. Total DPCs were isolated by RADAR and visualized by Flamingo protein gel staining. DPCs were analyzed by western blot for the indicated post-translational modifications (PTMs). Double-stranded DNA (dsDNA) was used as a loading control to show that DPCs were isolated from the same amount of genomic DNA. (B) FA treatment causes SUMO foci formation. RPE-1 cells were treated with 1 mM FA for 10 min at 37°C. EdU was added 20 min before FA treatment in order to label dividing cells. After treatment, cells were pre-extracted, fixed, and immunostained with the indicated antibodies. Bottom panels indicate quantifications of the number of foci per nucleus. Foci were counted with ImageJ (200 nuclei), and statistical significance was calculated using an unpaired t test. Pink lines represent mean of distribution. UT, untreated; Ub, ubiquitin. (C) DPC-induced SUMO-1 foci partially co-localize with γH2AX. RPE-1 cells were treated with 1 mM FA for 10 min at 37°C. After treatment, cells were pre-extracted, fixed, and immunostained with the indicated antibodies. Right-hand side plot shows the Pearson’s coefficient for SUMO-1 and γH2Ax colocalization. The pink line represents the mean of distribution. See also Figure S1.

To gain insights into the ubiquitin and SUMO signals associated with DPC repair in proliferative mammalian cells, we analyzed the dynamics of both post-translational modifications (PTMs) on DPCs following exposure to the general DPC-inducing agent FA (Figures 1A and S1A). DPCs were rapidly formed upon a 10-min pulse with FA in a dose-dependent manner (Figures 1A and S1A). Longer incubation times with FA did not increase DPCs but rather decreased their amount, suggesting the activation of fast DPC repair mechanisms (Figure S1A). We observed that FA-induced DPCs underwent extensive modification by SUMO-1, SUMO-2/3, and ubiquitin (Figure 1A). Increasingly high FA concentrations decreased the number of S phase cells and, accordingly, 5-ethynyl-2′-deoxyuridine (EdU) incorporation (Figures S1B and S1C). This indicates that FA-induced damage interferes with DNA replication. FA can also form interstrand crosslinks (ICLs), which are resolved by the Fanconi anemia pathway (Ceccaldi et al., 2016). To rule out its involvement in our experimental setup, we monitored the ubiquitylation status of FANCD2, a recognized marker for activation of the Fanconi anemia pathway. In contrast to mitomycin C (MMC) and cisplatin (cis) treatment, known ICL-inducing agents, FA treatment did not induce mono-ubiquitylation of FAND2 (Figure S1D, upper panel). Moreover, MMC or cisplatin treatment neither increased SUMOylation nor induced DPCs (Figure S1D, lower panel, and S1E), further indicating that our experimental setup with FA detected ubiquitylation and SUMOylation signals specifically associated with DPC and not ICL formation.

We proceeded to test whether FA induced accumulation of these PTMs in specific nuclear structures (foci). Short FA pulses led to a 2-fold increase in the average number of SUMO-1 and SUMO-2/3 foci both in S phase (EdU^+^) and non-S phase (EdU^−^) RPE-1 cells (Figure 1B). Interestingly, unlike SUMO, ubiquitin did not accumulate at specific foci but rather in a pan-nuclear pattern (Figures 1B and S1F), thus distinguishing FA-induced damage from double-strand break (DSB)-associated ubiquitin signaling, which typically shows focal accumulation (Messick and Greenberg, 2009). Confocal microscopy showed a modest co-localization of the SUMO-1 foci with the general DNA damage marker γH2AX, suggesting that SUMO-1 might accumulate at FA-induced DNA damage sites (Figure 1C).

We conclude that FA treatment rapidly triggers ubiquitylation and SUMOylation on total DPCs; however, microscopy analysis revealed formation of SUMO, but not ubiquitin, foci. This suggests that SUMO marks the sites for signaling DPC damage and possibly for DPC repair.

### SUMO and ubiquitin are required for replication-coupled DPC repair

**Figure 2:**
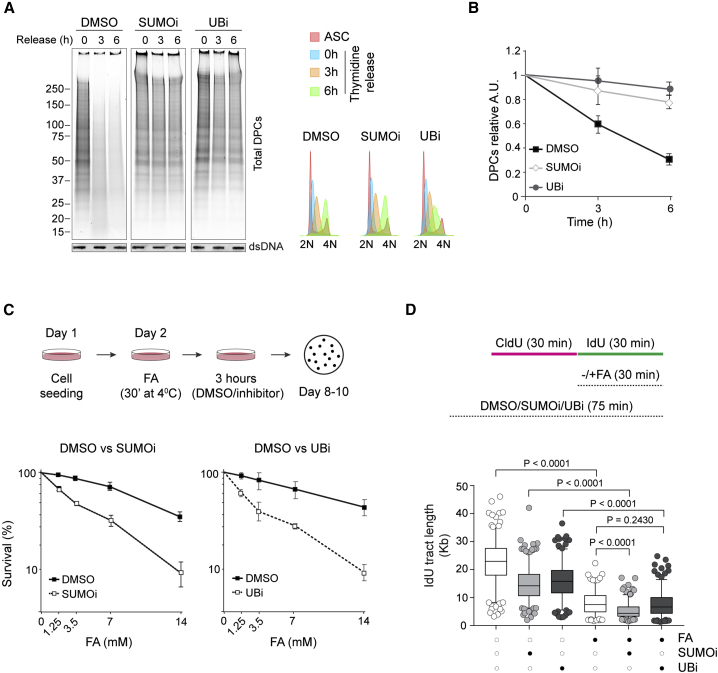
SUMO and ubiquitin are required for replication-coupled DPC repair (A) SUMOylation and ubiquitylation inhibition block DPC removal during S phase progression. HeLa cells were synchronized in G_1_/S with double thymidine block and released in the presence of DMSO, 25 μM 2-D08 (SUMOi), or 5 μM MLN7243 (UBi). Total DPCs were isolated by RADAR and detected by Flamingo protein gel staining. Slot blot with anti-dsDNA was used as a loading control. Right panel shows cell cycle distribution by fluorescence-activated cell sorting (FACS) analysis of the DNA content (propidium iodide). (B) Quantification of DPC removal for the experiment in (A). (C) SUMOylation and ubiquitylation inhibition sensitize cells to FA. Schematic of the survival assay protocol (upper panel). HeLa cells were exposed to the indicated concentrations of FA for 30 min at 4°C and let recover for 3 h in the presence of DMSO, 25 μM 2-D08 (SUMOi), or 5 μM MLN7243 (UBi). Colonies were allowed to grow for 8–10 days before fixation and counting (n = 2, mean ± SD). (D) SUMOylation and ubiquitylation inhibition reduce DNA replication speed. Box and whiskers plot for DNA combing analysis. HEK293 cells were allowed to incorporate chloro-deoxyuridine (CldU) for 30 min and iodo-deoxyuridine (IdU) for an additional 30 min in the presence of DMSO, 50 μM 2-D08 (SUMOi), or 5 μM MLN7243 (UBi). Where indicated, 450 μM FA was added for the duration of IdU incubation. The length of IdU tracts was measured with FiberVision software, and statistical significance was calculated using an unpaired t test (Mann-Whitney) (170–260 events). The graph is representative of two independent experiments.

DPCs are repaired during DNA replication (Duxin et al., 2014; Larsen et al., 2019; Vaz et al., 2016). To test whether SUMOylation and ubiquitylation are necessary for DPC repair in S phase, we monitored DPC removal in synchronous S phase HeLa cells treated with SUMOylation (2-D08) or ubiquitylation (MLN7243) inhibitors. Cells released from a double thymidine block into either ubiquitylation (UBi) or SUMOylation (SUMOi) inhibitors failed to repair DPCs during S phase progression (Figures 2A and 2B). This result indicates the requirement for both ubiquitin and SUMO in DPC repair during S phase.

We then addressed how treatments with SUMOi and UBi affects DNA replication and cell survival after FA treatment. Exposure to either inhibitor increases cellular sensitivity to FA (Figure 2C). Similarly, these inhibitors disturbed DNA replication fork progression in a DNA combing assay (Figure 2D). As previously reported, DNA track length was reduced by FA treatment (Halder et al., 2019; Mórocz et al., 2017; Vaz et al., 2016). Concomitant treatment with the SUMOylation inhibitor caused further reduction. The lack of a similar effect with the ubiquitylation inhibitor suggests that SUMO, but not ubiquitin, is required for replication progression in the short term.

Since replicating cells are the most sensitive to FA (Kumari et al., 2012; Vaz et al., 2016), this result supports our conclusion that SUMOylation and ubiquitylation are necessary for replication-coupled DPC repair.

### SUMOylated DPCs accumulate in SPRTN-depleted cells

**Figure 3:**
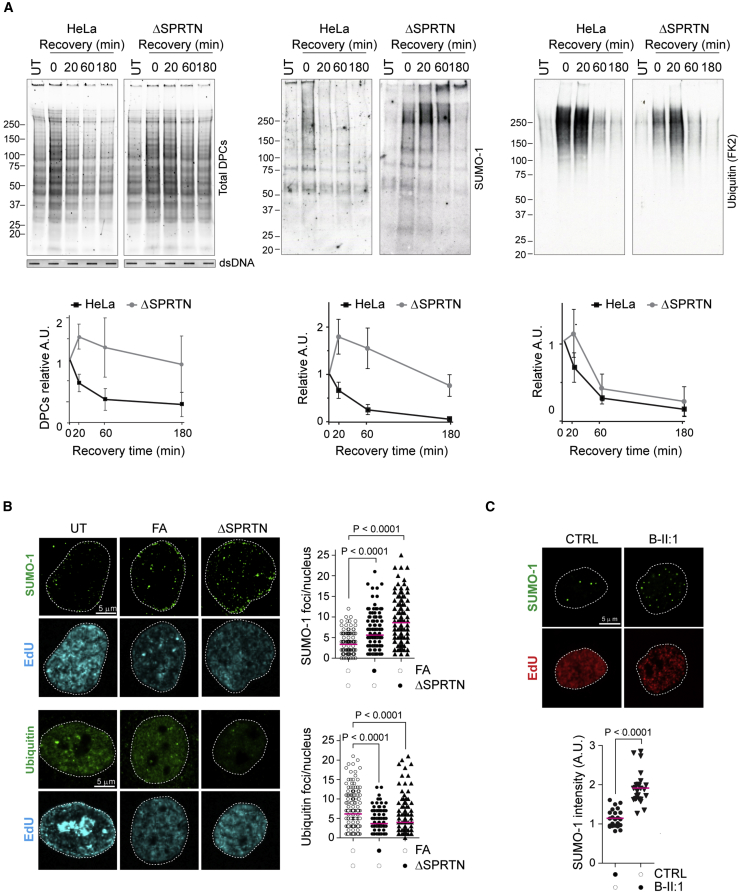
SUMOylated DPCs accumulate in SPRTN-depleted cells (A) Parental and ΔSPRTN HeLa cells were treated with 1.35 mM FA for 10 min at 37°C and allowed to recover for the indicated times. Total DPCs were isolated by RADAR and analyzed by western blot for the indicated PTMs. Graphs show the mean ± SEM of the relative signal from three independent experiments. (B) HeLa cells were treated with 1.35 mM FA for 10 min at 37°C. No FA was added to ΔSPRTN HeLa cells. Cells were pre-extracted, fixed, and immunostained with the indicated antibodies. Foci were counted from EdU-positive cells (200 nuclei) with ImageJ, and statistical significance was calculated using and unpaired t test. (C) Normal MRC5 (CTRL) and RJALS patient B-II:1 primary fibroblasts were pre-extracted, fixed, and immunostained with the indicated antibodies. The SUMO-1 signal was quantified in EdU-positive cells (25 nuclei) using ImageJ, and statistical significance was calculated using an unpaired t test. See also Figure S2.

We and others identified SPRTN as a DNA-dependent metalloprotease required for DPC proteolysis during DNA replication (Lopez-Mosqueda et al., 2016; Maskey et al., 2017; Mórocz et al., 2017; Stingele et al., 2016; Vaz et al., 2016). Therefore, we investigated whether DPC modifications are related to SPRTN-dependent repair. As previously reported, DPC removal was significantly affected in SPRTN-deficient cells (Figure 3A, far left panel) (Vaz et al., 2016). The DPC removal delay in SPRTN-haploinsufficient cells (ΔSPRTN) was not caused by cell cycle defects or replication arrest (Figures S2B and S2C). In parental cells, modified DPCs were removed within 60 min of recovery from FA treatment (Figures 3A and S2A). However, SPRTN deficiency caused persistence of SUMOylated DPCs (SUMO-2/3) and accumulation of high-molecular-weight SUMO-1 conjugates at later time points, while ubiquitylated DPCs were removed within 60 min (Figures 3A and S2A).

In addition, SPRTN-deficient cells (ΔSPRTN or siRNA) accumulated SUMO-1 foci (Figures 3B and S2D). Foci partially co-localized with γH2AX, suggesting their association with damage sites (Figure S2D). In contrast, and similar to FA treatment, ubiquitin foci were reduced in SPRTN-deficient cells (Figure 3B).

Lastly, we monitored SUMOylation in SPRTN-mutated cells from a Ruijs-Aalfs syndrome (RJALS) patient (Lessel et al., 2014). RJALS primary fibroblasts showed higher SUMO-1 intensity and foci number compared to control fibroblasts (Figure 3C), in agreement with our results in SPRTN-depleted cell lines.

Altogether, we show that DPC ubiquitylation is short-lived even when DPCs persist, as in SPRTN-depleted cells, indicating that ubiquitin is likely a transient signaling event. In contrast, modification by SUMO is more stable and correlates with DPC repair/SPRTN activity.

### SPRTN interacts with ubiquitin- and SUMO-modified proteins

**Figure 4:**
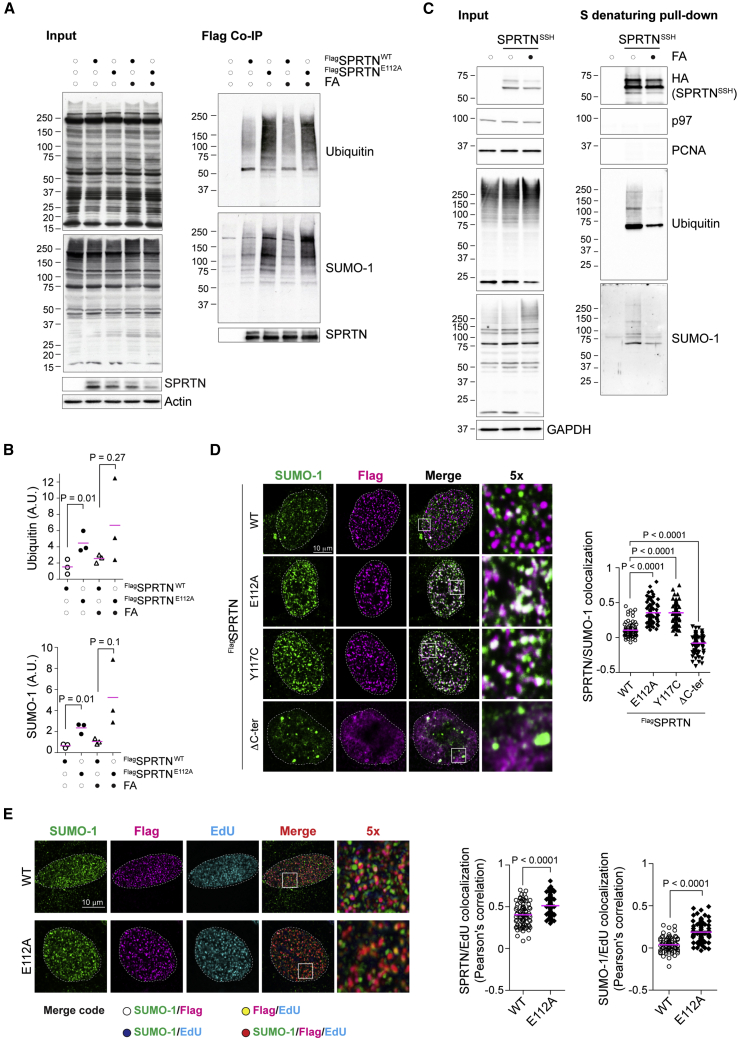
SPRTN interacts with ubiquitin- and SUMO-modified proteins (A) FLAG-SPRTN and FLAG-SPRTN-E112A were overexpressed in HEK293 cells and immunoprecipitated from total cell extracts under native conditions. Where indicated, cells had been treated with 1 mM FA for 1 h. The input and immunoprecipitates were analyzed by western blot for the indicated antibodies. Results are representative of three independent experiments. (B) Quantification for the experiment in (A). Plots indicate changes in the arbitrary units (A.U.). (C) SPRTN-SSH was overexpressed in HEK293 cells and purified from total cell extracts under denaturing conditions using Strep-Tactin Sepharose resin (S). Where indicated, cells had been treated with 1 mM FA for 1 h. The input and purified SPRTN were analyzed by western blot for the indicated antibodies. (D) U2OS cells overexpressing FLAG-SPRTN, FLAG-SPRTN-E112A, FLAG-SPRTN-Y117C, and the truncated version lacking the C-terminal half (ΔC-ter) were pre-extracted, fixed, and immunostained with the indicated antibodies. The plot indicates changes in Pearson’s correlation coefficient (50 nuclei). (E) U2OS cells overexpressing either the FLAG-SPRTN or the FLAG-SPRTN-E112A were labeled with EdU (10 min) and processed as in Figure 4D. Correlation coefficients were calculated as in Figure 4D (50 nuclei). See also Figure S3.

We tested whether SPRTN physically interacted with SUMO and ubiquitin conjugates after FA. We took advantage of the “trapping” effect of inactive protease mutants (Flynn et al., 2003; Westphal et al., 2012). We immunopurified FLAG-tagged SPRTN-WT or -E112A (a catalytic inactive mutant) from HEK293 cells under physiological conditions (150 mM NaCl). SUMO- and ubiquitin-modified proteins were detected in SPRTN’s immunoprecipitates (Figures 4A and 4B). Indeed, SPRTN protease-inactive variant (E112A) interacted with more ubiquitin and SUMO conjugates, strongly suggesting a substrate trapping effect. To rule out the possibility that modifications on SPRTN itself accounted for the PTM signals in the aforementioned immunoprecipitates, we isolated SPRTN-(Strep-tag)-(Strep-tag)-(HA tag) (SSH) under denaturing conditions (Figure 4C). As expected, here we could not detect p97 and PCNA, two known SPRTN interactors (Centore et al., 2012; Davis et al., 2012; Ghosal et al., 2012; Juhasz et al., 2012; Machida et al., 2012; Mosbech et al., 2012), which instead were readily detected in SPRTN’s native immunoprecipitates (Figure S4A). SPRTN appeared to be modified by both SUMO and ubiquitin, but after FA treatment these modifications decreased, in line with previously published data (Zhao et al., 2021). Thus, the large majority of the SUMO and ubiquitin signals in SPRTN immunoprecipitates come from substrates and binding proteins, especially after FA treatment when SPRTN engages in DPC proteolysis.

To complement these results we analyzed co-localization of different SPRTN variants with SUMO-1 foci in U2OS cells by confocal microscopy (Figure 4D) and employed Pearson’s correlation coefficient to quantify the co-localization of the two signals. While a modest average correlation of 0.1 was detected between the wild-type SPRTN and SUMO-1, a significant increase to 0.4 was observed in cells expressing the protease-inactive variant E112A, further confirming a trapping effect (Figure 4D). Similar to SPRTN-E112A, one of the RJALS patient variants (Y117A), also proteolytically defective (Lessel et al., 2014; Vaz et al., 2016), co-localized with SUMO-1 foci (0.4). No correlation was observed in cells expressing another RJALS patient SPRTN variant lacking the C-terminal half (ΔC-ter, Lys241AsnfsX8) (Lessel et al., 2014), suggesting a possible involvement of SPRTN’s C-terminal part in mediating re-location to SUMO foci. The defective localization of SPRTN’s patient variants could account for the etiology of RJALS syndrome. We also noticed an increase in the co-localization between SPRTN/SUMO and EdU in cells expressing the inactive SPRTN variant E112A (Figure 4E), suggesting that, at least in part, SPRTN colocalizes with SUMO-1 conjugates at sites of active DNA replication, likely during replication-dependent proteolysis.

We next tested whether substrate modification by ubiquitin/SUMO is required for SPRTN-dependent proteolysis *in vitro*. As a substrate, we chose Topo-1 isolated under denaturing conditions from HEK293 cells (Figure S3). During CPT treatment, Topo-1 becomes covalently attached to DNA (Topo-1-cc) and modified by ubiquitin and SUMO-1 in a dose-dependent manner (Desai et al., 2003; Fielden et al., 2020; Lin et al., 2008; Mao et al., 2000; Sun et al., 2020) (Figure S3A). Our purification procedure successfully isolated unmodified and modified Topo-1 from chromatin (Figure S3A). When these species were incubated *in vitro* with purified SPRTN, all Topo-1 forms (unmodified and modified) were cleaved (Figure S3B). More importantly, the unmodified Topo-1 from DMSO- or CPT-treated cells (asterisk) was processed to a similar extent, and the cleavage products were seemingly comparable in size and intensity (arrowheads). This suggests that the highly modified Topo-1 species from CPT-treated cells are not preferred over unmodified Topo-1. The GFP antibody specifically detected YFP-Topo-1 fragments, and no cross-reactivity with recombinant SPRTN was observed (Figure S3C).

Thus, in response to FA treatment SPRTN interacts with ubiquitylated and SUMOylated substrates; however, these two modifications do not directly favor proteolysis over unmodified substrates, at least *in vitro*.

### Ubiquitylation is required for SPRTN-mediated repair

**Figure 5:**
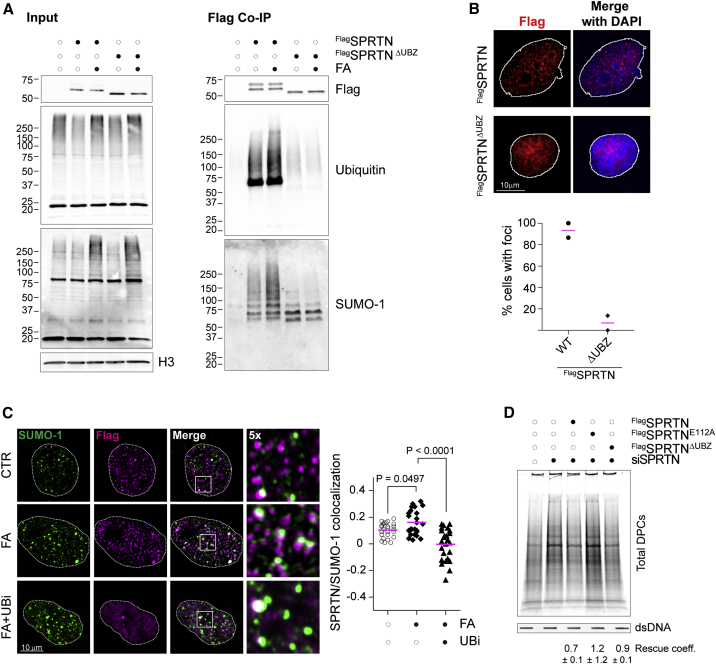
Ubiquitylation is required for SPRTN-mediated repair (A) FLAG-SPRTN and FLAG-SPRTN ΔUBZ were overexpressed in HEK293 cells for 15 h and immunoprecipitated from total cell extracts under native conditions. Where indicated, cells had been treated with 1 mM FA for 1 h. The input and immunoprecipitate were analyzed by western blot for the indicated antibodies. Results are representative of three independent experiments. (B) U2OS cells overexpressing either FLAG-SPRTN or FLAG-SPRTN ΔUBZ were treated with 1 mM FA. One hour after treatment cells were pre-extracted, fixed, and immunostained with anti-FLAG antibody. The graph reports the percentage of cells showing SPRTN foci (n = 2, more than 30 FLAG-SPRTN-transfected cells/condition/experiment). (C) U2OS cells expressing FLAG-SPRTN were treated with 1 mM FA alone or in the presence of 5 μM MLN7243 (UBi). One hour after treatment cells were pre-extracted, fixed, and immunostained with the indicated antibodies. The plot shows changes in Pearson’s correlation coefficient (25 nuclei/condition). (D) HEK293 cells were depleted of SPRTN for 3 days. FLAG-SPRTN, FLAG-SPRTN-E112A, or FLAG-SPRTN ΔUBZ were overexpressed for 15 h before harvesting the cells for DPC isolation. Total DPCs were visualized by Flamingo protein gel staining. Slot blot with anti-dsDNA was used as a loading control. The rescue coefficient (Rescue coeff.) was calculated as the ratio between the total DPCs in the relevant lane and the total DPCs in si*SPRTN* cells and averaged from two independent experiments (mean ± SD). See also Figures S4 and S5.

So far, we have demonstrated that both ubiquitylation and SUMOylation are necessary for DPC repair, and that SPRTN binds and processes DPCs decorated with ubiquitin and SUMO. To gain insights into how ubiquitin and SUMO affect SPRTN-dependent DPC proteolysis in cells, we focused on SPRTN’s UBZ motif, which mediates its direct interaction with ubiquitin (Centore et al., 2012; Davis et al., 2012; Juhasz et al., 2012; Machida et al., 2012; Mosbech et al., 2012). We asked whether the UBZ motif is needed for recruitment of SPRTN to FA-induced damage sites. First, we confirmed that a SPRTN variant bearing a deletion of the UBZ motif (ΔUBZ) lost its ability to bind ubiquitin conjugates (Figure 5A), while still retaining binding to p97 and PCNA (Figure S4A). This indicates that the UBZ deletion does not significantly alter SPRTN’s structure. SUMO conjugates were also depleted from the SPRTN ΔUBZ pull-down, suggesting that the interacting ubiquitylated species are co-modified with SUMO (Figure 5A). Second, we analyzed the recruitment of SPRTN ΔUBZ to the chromatin and FA-induced nuclear foci. SPRTN’s levels are expected to increase at the chromatin after FA exposure (Stingele et al., 2016; Zhao et al., 2021) (Figures S4B–S4D). SPRTN ΔUBZ was able to localize at the chromatin (Figure S4B) (Stingele et al., 2016); however, in contrast to SPRTN WT, it failed to form nuclear foci after FA treatment (Figure 5B) (90% versus 9% of cells displayed nuclear foci). This is in line with previous evidence showing that SPRTN fails to form nuclear foci following FA in the presence of the ubiquitylation inhibitor MLN7243 (Borgermann et al., 2019). We recapitulated this result and found that SPRTN and SUMO-1 foci showed reduced colocalization in MLN7243-treated cells (UBi) (Figure 5C). At the chromatin, UBi caused accumulation of SPRTN regardless of FA treatment (Figure S4C). This is expected, since UBi causes accumulation of SPRTN’s de-ubiquitylated form, which is more stable (Figure S4C) (Zhao et al., 2021). Conversely, neither SUMOylation inhibition nor *UBC9* silencing affected SPRTN recruitment to chromatin or its de-ubiquitylation/activation after FA treatment (Figures S4C and S4D). These results indicate that ubiquitylation and SPRTN’s UBZ motif are not needed for chromatin relocation per se, but are necessary for recruitment to repair foci after FA treatment. This would lead to the hypothesis that SPRTN’s UBZ mutant variant is non-functional. Indeed, overexpression of SPRTN ΔUBZ in *SPRTN*-depleted cells did not fully rescue DPC levels, when compared to overexpression of SPRTN WT (Figure 5D), consistent with previously published data (Larsen et al., 2019). Overall, these results indicate that the ubiquitylation signal induced by FA treatment is recognized by SPRTN’s UBZ motif for its proper localization to DPC repair sites and consequently DPC repair.

Ubiquitylation can lead to proteasomal degradation. In light of recent reports showing proteasome-dependent DPC repair mechanisms (Larsen et al., 2019; Sparks et al., 2019; Sun et al., 2020), we asked whether the 26S proteasome might also function in ubiquitin-dependent DPC proteolysis. Total DPC removal kinetics were not delayed by the proteasome inhibitor MG132 following recovery from a short pulse with FA (Figures S5A and S5B). Similarly, recovery of *SPRTN*-depleted cells in the presence of MG132 only slightly, but not significantly, delayed total DPC repair (Figures S5A and S5B), while still causing accumulation of cellular ubiquitin conjugates (Figure S5C). Proteasome inhibition slightly affected repair of Topo-1-ccs, a specific and CPT-induced type of DPC (Figure S5D). We therefore conclude that the proteasome has a negligible role in DPC removal under our experimental conditions, and that transient ubiquitylation is predominantly a signal for SPRTN recruitment and SPRTN-dependent proteolysis.

### SUMO suppresses HR at DPC-induced DNA damage sites

SUMOylation is essential for DPC repair and DNA replication fork progression over FA-induced lesions. Our data (Figure 3) suggest a relationship between SPRTN-mediated proteolysis and FA-induced SUMOylation. However, we were not able to conclusively prove a physical interaction between SPRTN and SUMO-1 or SUMO-2/3 using recombinant proteins (data not shown). Thus, we could not directly assess the link between SPRTN proteolysis and SUMOylation in cells as we did for SPRTN ΔUBZ and ubiquitin.

**Figure 6:**
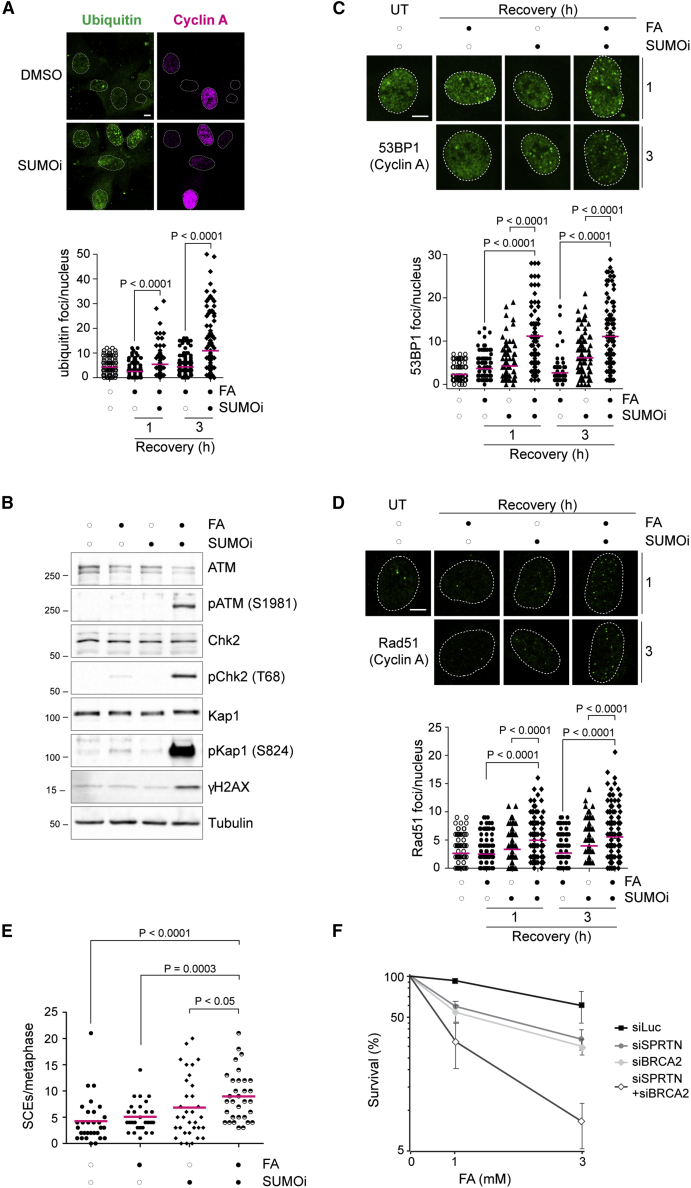
SUMO suppresses homologous recombination at DPC-induced DNA damage sites (A) RPE-1 cells were treated with 1 mM FA for 20 min at 4°C and allowed to recover for the indicated times in the presence of DMSO or 25 μM 2-D08 (SUMOi). After treatment, cells were fixed and immunostained with the indicated antibodies. (Top) Representative image at 3 h after treatment. (Bottom) Graphic representation of the number of foci per nucleus (150 nuclei) counted with ImageJ. Statistical significance was calculated using an unpaired t test. Scale bar, 5 μm. (B) RPE-1 cells were treated with 1 mM FA for 1 h, in combination with DMSO or 25 μM 2-D08. Total cell extracts were prepared and analyzed by western blot for the indicated proteins. Results are representative of three independent experiments. (C) RPE-1 cells were treated with 800 μM FA for 10 min at 37°C and allowed to recover for the indicated times in the presence of DMSO or 25 μM 2-D08 (SUMOi). After treatment, cells were fixed and immunostained with the indicated antibodies. (Upper) Representative images. (Lower) Graphic representation of the number of 53BP1 foci per nucleus in cyclin A-positive cells (100 nuclei) counted using ImageJ. Statistical significance was calculated using an unpaired t test. Scale bar, 5 μm. (D) RPE-1 cells were treated with 800 μM FA for 10 min at 37°C and allowed to recover for the indicated times in the presence of DMSO or 25 μM 2-D08 (SUMOi). After treatment, cells were fixed and immunostained with the indicated antibodies. (Upper) Representative images. (Lower) graphical representation of the number of Rad51 foci per nucleus in cyclin A-positive cells (190 nuclei) counted using ImageJ. Statistical significance was calculated using an unpaired t test. Scale bar, 5 μm. (E) HeLa cells were grown for 48 h in the presence of BrdU, exposed to 450 μM FA for 10 min at 37°C with 10 μM 2-D08 (SUMOi) or DMSO, and allowed to recover for 16 h in the presence of colcemid with DMSO or 10 μM 2-D08. Metaphase spreads were stained and SCE events counted from at least 30 nuclei. Results are representative of two independent experiments. (F) Depleted cells were exposed to the indicated concentrations of FA for 15 min at 37°C. Colonies were allowed to grow for 8–10 days before fixation and counting. Graphical representation of the survival fraction from two independent experiments (mean ± SD). See also Figure S6.

To understand the role of SUMOylation, we turned to the SUMOylation inhibitor (2-D08). We analyzed the interplay between SUMO and ubiquitin at DPC-induced damage sites. SUMOylation-deficient cells showed progressive build-up of ubiquitin foci following a 3 h recovery from a short FA pulse, with an increase of up to 3-fold (Figure 6A). Co-localization of ubiquitin with γH2AX foci in SUMOylation-defective cells indicates that ubiquitin foci formed at FA-induced damage sites (Figure S6A). Interestingly, SUMOylation inhibition or SPRTN inactivation (ΔSPRTN) led to a robust increase in the ubiquitin signal at DNA damage sites following recovery from UV laser micro-irradiation, another source of DPCs (Pashev et al., 1991; Shetlar et al., 1984) (Figures S6B and S6C). These results suggest that SUMOylation and SPRTN suppress excessive ubiquitination at DPC-induced DNA damage sites.

In yeast, SUMO suppresses recombinogenic events (Branzei et al., 2006). In yeast and human cells, HR is protective toward DPC-induced toxicity (de Graaf et al., 2009; Nakano et al., 2009) and is active in S phase. HR can overcome DPC-induced damage when prolonged replication fork stalling causes fork collapse and DSB formation (Cortez, 2015). Therefore, we asked (1) whether the ubiquitylation signal in the presence of the SUMOylation inhibitor is associated with HR, a pathway known to be heavily dependent on ubiquitin signaling (Smeenk and Mailand, 2016); and (2) whether DPC persistence in SUMOylation-defective cells switches the DPC repair pathway from SPRTN-dependent proteolysis to HR. Indeed, following treatment with FA, SUMOi-treated RPE-1 cells showed an increase in phospho-ATM, phospho-H2Ax (γH2Ax), phospho-CHK2, and phospho-KAP1, well-defined markers of DSBs (Figure 6B).

To further show that FA treatment causes DSBs in SUMOylation-deficient cells, we monitored Rad51 and 53BP1 foci, two well-recognized markers for DSB formation, by immunofluorescence microscopy. Cells recovering from FA treatment in the presence of SUMOylation inhibitor showed a 4-fold increase in the average number of 53BP1 foci (Figure 6C) and a 2-fold increase in the average number of Rad51 foci per nucleus (Figure 6D). Finally, we scored sister chromatid exchange (SCE) events, which result from HR activity. Cells treated with FA and allowed to recover in the presence of the SUMOylation inhibitor had more SCEs than did cells exposed to any of the individual treatments (Figures 6E and S6D).

Collectively, this set of data supports a role for SUMO in suppressing DSB formation during FA-induced damage, and consequent activation of an ubiquitin-dependent, recombinogenic pathway acting as an alternative to SPRTN proteolysis in DPC repair. Importantly, two additional pieces of evidence confirmed that HR is a back-up for DPC repair in SPRTN-deficient cells. First, depletion of the HR factor BRCA2 further sensitized *SPRTN*-depleted cells to FA (Figure 6F). Second, *SPRTN* depletion by siRNA in *BRCA2*-deficient cells inhibited growth and caused cell death, demonstrating synthetic lethality between the two DPC repair pathways (Figure S6E).

Overall, these results show that the SPRTN-SUMO axis protects cells from DPC-induced DSBs and prevents HR activation, which can lead to chromosomal instability.

## Discussion

Our results provide insights into the post-translational signaling mechanisms associated with replication-coupled DPC proteolysis. We demonstrate that SUMOylation and ubiquitylation promote DPC clearance, allowing unperturbed DNA replication fork progression and preventing DPC-induced cytotoxicity. We show that ubiquitylation is necessary for SPRTN localization at repair foci and for DPC repair. Inactivation of the ubiquitin-SPRTN-SUMO axis leads to DSB formation, HR activation, and, potentially, deleterious genomic rearrangements.

SPRTN and its yeast ortholog Wss1 are pleiotropic proteases that cleave various DNA-binding proteins *in vitro* (Mórocz et al., 2017; Stingele et al., 2014, 2016; Vaz et al., 2016). In cells, their activities are regulated to avoid uncontrolled cleavage of nuclear proteins (Fielden et al., 2018; Ruggiano and Ramadan, 2021b; Stingele et al., 2015). Strong transient overexpression of SPRTN has indeed been found to be toxic (Lessel et al., 2014). Considering that SPRTN travels with the replication fork (Maskey et al., 2017; Vaz et al., 2016), replisome components are at particularly high risk of potentially undesired cleavage. Therefore, the labeling of SPRTN substrates with PTMs arises as a plausible regulatory mechanism to prevent uncontrolled proteolysis.

SPRTN has a UBZ motif for direct binding to ubiquitin. Although DPC ubiquitylation is transient (Figure 3A), it is plausible that SPRTN interacts directly with its substrates. The extent of the modification is unclear, and might involve proteins other than the DPCs themselves. In such case, we envision that other regulatory modes (e.g., the single-stranded DNA [ssDNA]-double-stranded DNA [dsDNA] junction) will restrict SPRTN activity to DPCs preserving functional proteins (Zhao et al., 2021). In line with this, our cell fractionation experiments suggest additional modes of regulation. The UBZ motif mutant is recruited to the chromatin but remains incapable of localizing to foci (Figures 5B and S4) (Stingele et al., 2016). This suggests that relocation to chromatin does not simply depend on interaction with ubiquitin conjugates after FA, but other recruitment/regulatory mechanisms must be in place (e.g., accessory proteins or other PTMs on SPRTN).

Studies in a *Xenopus* cell-free system implicated the proteasome in the degradation of ubiquitylated DPCs (Larsen et al., 2019). Nonetheless, in our system proteasome inhibition does not affect the repair of total DPCs (Figure S5A), largely excluding this proteolytic pathway from replication-coupled repair in cells. These differences may be due to the excess of proteins in *Xenopus* egg extracts compared to cell-based systems and/or the lower complexity of the plasmid-DPC investigated in *Xenopus* cell-free extracts compared to chromatin in the cell. We do observe a slight, although not significant, defect in DPC clearance when the proteasome is blocked in SPRTN-depleted cells (Figure S5), suggesting that the proteasome could come into play when DPC formation exceeds SPRTN’s repair capacity. Other reports implicated the proteasome in Topo1/2-cc proteolysis (Desai et al., 1997; Lin et al., 2008; Mao et al., 2001). We observed a slight MG132-dependent delay in the repair of Topo-1cc generated with a low dose of CPT (Figure S5D). However, proteasome inhibition might cause Topo-1cc accumulation only when cells are exposed to high doses of CPT (20 μM or higher) (Desai et al., 1997; Interthal and Champoux, 2011; Lin et al., 2008; Mao et al., 2001; Sordet et al., 2008) rather than to the low and clinically relevant doses (50 nM) used in our experimental setup.

Besides DPC proteolysis, recombination-dependent pathways can lead to DPC damage tolerance under certain circumstances. Prolonged fork stalling due to DPCs can lead to fork breakage, thus setting the ground for HR involvement (Stingele and Jentsch, 2015). We observed the occurrence of a strong ubiquitin signal when SUMOylation is inhibited, and recruitment and regulation of several HR proteins, including RAP80-Abraxas, BRCA1, and Rad51, heavily rely on ubiquitin (Schwertman et al., 2016). Moreover, we show that SUMOylation inhibition increases DSB signaling (Figure 6B), Rad51 and 53BP1 foci (Figures 6C and 6D), and SCEs, which result from HR activity (Figure 6E). Collectively, these data indicate that SUMOylation suppresses HR in the context of FA-induced DPC repair. The synthetic lethality of *BRCA2*- and *SPRTN*-depleted cells further indicates that HR and DPC proteolysis via SPRTN work in parallel, alternative pathways. Considering that HR can lead to chromosomal rearrangements (Guirouilh-Barbat et al., 2014), SPRTN proteolysis seems a safer choice.

Our results establish a correlation between SPRTN and DPC-induced SUMOylation: (1) SUMO accumulates in nuclear foci and on DPCs in *SPRTN*-deficient cells (Figures 3 and S2); (2) SPRTN foci colocalize with SUMO-1 foci (Figures 4 and 5); and (3) SPRTN interacts with SUMO conjugates in an UBZ-dependent manner (Figures 4 and 5). However, it remains unclear whether SUMOylation fosters DPC proteolysis directly or indirectly. In our hands, recombinant SPRTN does not bind SUMO *in vitro* (data not shown), and *in vitro* proteolysis of a model substrate occurs regardless of its PTM status (Figure S3). Thus, an indirect mechanism seems plausible.

Our results are in line with previous observations made in yeast, showing that SUMOylation counteracts recombinogenic events at damaged replication forks (Branzei et al., 2006). However, how SUMO regulates DPC proteolysis in mammalian cells needs to be elucidated.

### Limitations of study

The identification of the E3 SUMO ligase could help to strengthen the role of SUMO in DPC repair. Targeted depletion of the E3 (e.g., via auxin-inducible degrons) will be a cleaner system than the use of SUMOylation inhibitors. Previous studies on SPRTN have focused on the function of ubiquitin in DPC proteolysis; we expect that our study will foster research on SUMOylation in replication-coupled DPC repair.

## STAR★Methods

### Key resources table

**Table:** 

REAGENT or RESOURCE	SOURCE	IDENTIFIER
**Antibodies**		

Rabbit anti-SUMO-1	Abcam	Cat#ab11672; RRID:AB_298480
Rabbit anti-SUMO-2/3	Cell Signaling	Cat#4971; RRID:AB_2198425
Mouse anti-ubiquitin [FK2]	Enzo Life Sciences	Cat#BML-PW8810; RRID:AB_10541840
Rabbit anti-SPRTN	Atlas	Cat#HPA025073; RRID:AB_1847695
Rabbit anti-SPRTN	In house	N/A
Mouse anti-Topoisomerase-1	Merck Millipore	Cat#MABE1084; RRID:AB_2756354
Rabbit anti-FANCD2	Novus Biologicals	Cat#NB100-182; RRID:AB_10002867
Mouse anti-γH2AX	Merck Millipore	Cat#05-636; RRID:AB_309864
Rabbit anti-γH2AX	Novus Biologicals	Cat#NB100-2280; RRID: AB_10000580
Rabbit anti-53BP1	Santa Cruz Biotechnology	Cat#sc-22760; RRID:AB_2256326
Rabbit anti-phospho-ATM (S1981)	Abcam	Cat#ab81292; RRID:AB_1640207
Rabbit anti-phospho-Chk2 (T68)	Cell Signaling	Cat#2661; RRID:AB_331479
Rabbit anti-phospho-Kap1 (S824)	Abcam	Cat#ab70369; RRID:AB_1209417
Mouse anti-ATM	Sigma-Aldrich	Cat#A1106; RRID:AB_796190
Rabbit anti-Chk2	Cell Signaling	Cat#2662; RRID:AB_2080793
Rabbit anti-Kap1	Abcam	Cat#ab10484; RRID:AB_297223
Rabbit anti-GFP	Abcam	Cat#ab290; RRID:AB_303395
Mouse anti-Flag	Sigma-Aldrich	Cat#F1804; RRID:AB_262044
Rabbit anti-Flag	Sigma-Aldrich	Cat#F7425; RRID:AB_439687
Rat anti-HA [3F10]	Roche	Cat# 11867423001; RRID:AB_390918
Rabbit anti-cyclin A	Santa Cruz Biotechnology	Cat#sc-751; RRID:AB_631329
Mouse anti-cyclin A2	Abcam	Cat#ab38; RRID:AB_304084
Rabbit anti-p97	Proteintech	Cat#10736-1-AP; RRID:AB_2214635
Mouse anti-PCNA [PC10]	Abcam	Cat#ab29; RRID:AB_303394
Rabbit anti-Ubc9	Abcam	Cat#ab75854; RRID:AB_1310787
Mouse anti-Lamin A/C	Cell Signaling	Cat#4777; RRID:AB_10545756
Rabbit anti-Histone 3	Abcam	Cat#ab1791; RRID:AB_302613
Rabbit anti-GAPDH	Proteintech	Cat#10494-1-AP; RRID:AB_2263076
Mouse anti-αtubulin	Sigma-Aldrich	Cat#T6199; RRID:AB_477583
Mouse anti-βactin	ThermoFisher Scientific	Cat#AM4302; RRID:AB_437394
Mouse anti-Vinculin	Abcam	Cat#ab18058; RRID:AB_444215
Mouse anti-dsDNA	Abcam	Cat#ab27156; RRID:AB_470907
Rat anti-5-bromo-2′-deoxyuridine (BrdU) [BU1/75 (ICR1)]	Abcam	Cat#ab6326; RRID:AB_305426
Mouse anti-5-bromo-2′-deoxyuridine (BrdU)	BD Biosciences	Cat#347580; RRID:AB_400326
Rabbit anti-mouse IgG-horseradish peroxidase (HRP)	Sigma-Aldrich	Cat#A9044; RRID:AB_258431
Goat anti-rabbit IgG-HRP	Sigma-Aldrich	Cat#A9169; RRID:AB_258434
Goat anti-rat IgG (Cy5®)	Abcam	Cat#Ab6565; RRID:AB_955063
Goat anti-mouse IgG (Cy3.5®)	Abcam	Cat#Ab6946; RRID:AB_955045
Goat anti-rabbit IgG-Alexa Fluor Plus 488	ThermoFisher Scientific	Cat#A32731; RRID:AB_2633280
Goat anti-rabbit IgG-Alexa Fluor Plus 594	ThermoFisher Scientific	Cat#A32740; RRID:AB_2762824
Goat anti-mouse IgG-Alexa Fluor 594	ThermoFisher Scientific	Cat#R37121; RRID:AB_2556549
Donkey anti-mouse IgG-Alexa Fluor 568	ThermoFisher Scientific	Cat#A10037; RRID:AB_2534013

**Bacterial and virus strains**		

*E.coli* Subcloning efficiency DH5α competent cells	ThermoFisher Scientific	Cat#18265-017
*E.coli* Rosetta 2 (DE3)	Novagen	Cat#71405-3

**Biological samples**		

RJALS patient B-II:1 primary fibroblasts	Lessel et al., 2014	N/A
RJALS patient lymphoblastoid cell lines	Lessel et al., 2014	N/A

**Chemicals, peptides, and recombinant proteins**		

Formaldehyde (FA) solution	Fisher Scientific	Cat#F/1501/PB08
Mitomycin C (MMC)	Abcam	Cat#ab120797
Cisplatin	Sigma-Aldrich	Cat#P4394
Camptothecin (CPT)	Selleckchem	Cat#S1288
MLN7243	Chemietek	Cat#CT-M7243
ML-792	MedChemExpress	Cat#HY-108702
2-D08	Sigma-Aldrich	Cat#SML1052
MG132	Sigma-Aldrich	Cat#474790
BrdU	Sigma-Aldrich	Cat#B5002
CldU	Sigma-Aldrich	Cat#C-6891
IdU	Sigma-Aldrich	Cat#I-7125
EdU	Santa Cruz Biotechnology	Cat#sc-284628
Hoechst 33258	Sigma-Aldrich	Cat#B2883
DAPI staining solution	Abcam	Cat#ab228549
Propidium Iodide	Sigma-Aldrich	Cat#P4864
KaryoMAX Colcemid solution	ThermoFisher Scientific	Cat#15212012
Proteinase K	New England BioLabs	Cat#P8107S
Benzonase	Merck Millipore	Cat#71205-3
RNase A	ThermoFisher Scientific	Cat#EN0531
Flag® peptide	Sigma-Aldrich	Cat#F3290
Recombinant SPRTN wt	In house	N/A
Recombinant SPRTN E112A	In house	N/A

**Critical commercial assays**		

Quant-iT PicoGreen dsDNA assay kit	ThermoFisher Scientific	Cat#P11496
Click-iT® EdU Cell Proliferation Assay kit	ThermoFisher Scientific	Cat#C10632
Flow Cytometry Assay kit	ThermoFisher Scientific	Cat#C10425
Flamingo Fluorescent Protein Gel Stain	Bio-Rad	Cat#161-0490
FiberPrep® DNA extraction Kit	Genomic Vision	Cat#EXTR-001

**Deposited data**		

All raw data deposited on Mendeley	This paper	https://doi.org/10.17632/srx6r55y4s.1

**Experimental models: Cell lines**		

Human: cervical carcinoma HeLa cells (female)	ATCC®	CCL-2; RRID:CVCL_0030
Human: ΔSPRTN HeLa cells	Ramadan lab; Vaz et al., 2016	N/A
Human: embryonic kidney HEK293 cells (female)	ATCC®	CRL-1573, RRID:CVCL_0045
Human: epithelial hTERT RPE-1 (female)	ATCC®	CRL-4000
Human: osteosarcoma U-2 OS cells (female)	ATCC®	HTB-96; RRID:CVCL_0042
Human: colon epithelium DLD-1 cells (male)	ATCC®	CCL-221
Human: colon epithelium BRCA2^−/−^ DLD-1 cells (male)	Horizon Discovery	HD 105-007; RRID:CVCL_HD57

**Experimental models: Organisms/strains**		

N/A		

**Oligonucleotides**		

siRNA targeting sequence (Luciferase): CGUACGCGGAAUACUUCGA	This study	N/A
siRNA targeting sequence (UBC9): GUGGCUGUCCCAACAAAAA	This study	N/A
siRNA targeting sequence (SPRTN #1): GUCAGGAAGUUCUGGUUAA	Ramadan lab; Vaz et al., 2016	N/A
siRNA targeting sequence (SPRTN #2): CACGAUGAGGUGGAUGAGUAU	Ramadan lab; Vaz et al., 2016	N/A
siRNA targeting sequence (SPRTN #3): AGCCAAUAUAACGGUAUACCA	Ramadan lab; Vaz et al., 2016	N/A
ON-TARGETplus siRNA reagents - Human BRCA2	Dharmacon	Cat#J-003462-05; Lot#161117

**Recombinant DNA**		

pcDNA3.1 Flag-SPRTN wt	Lessel et al., 2014	N/A
pcDNA3.1 Flag-SPRTN E112A	Ramadan lab; Vaz et al., 2016	N/A
pcDNA3.1 Flag-SPRTN Y117C	Ramadan lab	N/A
pcDNA3.1 Flag-SPRTN ΔC-ter (Lys241AsnfsX8)	Ramadan lab	N/A
pcDNA3.1 Flag-SPRTN ΔUBZ (Δ452-482aa)	This study	N/A
pNIC-ZB SPRTN wt	Ramadan lab; Vaz et al., 2016	N/A
pNIC-ZB SPRTN E112A	Ramadan lab; Vaz et al., 2016	N/A
YFP-Topoisomerase 1	Sherif El-Khamisy lab; Vaz et al., 2016	N/A
pcDNA5/FRT/TO SPRTN-SSH	Ramadan lab	N/A

**Software and algorithms**		

ImageJ	National Institutes of Health	https://imagej.nih.gov/ij/
ImageLab software	Biorad	https://www.bio-rad.com/en-uk/product/image-lab-software?ID=KRE6P5E8Z
GraphPad	Prism	https://www.graphpad.com/scientific-software/prism/
Biorender	Biorender	https://biorender.com/
FlowJo	BD	https://www.flowjo.com/solutions/flowjo
FiberStudio® v0.15	Genomic Vision	N/A
GelCount	Oxford Optronix	https://www.oxford-optronix.com/gelcount-cell-colony-counter

**Other**		

BD FACSCalibur flow cytometer	BD Biosciences	N/A
FiberVision® platform	Genomic Vision	N/A
Lipofectamine RNAiMAX	ThermoFisher Scientific	Cat#13778150
Lipofectamine® 3000	ThermoFisher Scientific	Cat#L3000015
Fugene**®** HD transfection reagent	Promega	Cat#E2311
Fetal Bovine Serum	Sigma-Aldrich	Cat#F9665
Dulbecco’s Modified Eagle’s Medium (DMEM)	Sigma-Aldrich	Cat#D6429
Pen/Strep solution	Sigma-Aldrich	Cat#P4333
optimal hypotonic solution	Genial Helix	Cat#GGS-JL005A
Anti-FLAG**®** M2 Affinity Gel	Millipore	Cat#A2220
Anti-FLAG**®** M2 Magnetic beads	Millipore	Cat#8823
Strep-Tactin**®** Sepharose**®** resin	IBA Lifesciences	Cat#2-1201-010
GFP-trap®	Chromotek	Cat#gta-20
ProLong Gold Antifade Mountant	ThermoFisher Scientific	Cat#P36930
ProLong Diamond Antifade Mountant with DAPI	ThermoFisher Scientific	Cat#P36971
Crystal Violet	TCS Biosciences	Cat#HD1295
Nitrocellulose membrane	Amersham	Cat#GE10600008
Polyvinylidene fluoride (PVDF)	Bio-Rad	Cat#1620177
Nylon membrane	GE Healthcare	Cat#RPN303N

### Resource availability

#### Lead contact

Further information and requests for resources and reagents should be directed to and will be fulfilled by the lead contact, Kristijan Ramadan (kristijan.ramadan@oncology.ox.ac.uk).

#### Materials availability

Plasmids generated in this study are available from the lead contact with a completed materials transfer agreement.

### Experimental models and subject details

#### Cell lines

HeLa, U2OS, RPE-1 and HEK293 cell lines were obtained from the American Type Culture Collection (ATCC). All cell lines were grown in complete medium supplemented with 10% FBS.

### Method details

#### Cell culture

HEK293, HeLa, RPE-1 and U2OS cells were grown in Dulbecco’s Modified Eagle’s medium (DMEM, Sigma-Aldrich) supplemented with 10% fetal bovine serum (Sigma-Aldrich) and 100 I.U./mL penicillin - 0.1 mg/mL streptomycin (Sigma-Aldrich) at 37°C in a humidified incubator with 5% CO_2_, and tested for mycoplasma contamination. CRISPR partial knockout ΔSPRTN HeLa cells (Vaz et al., 2016) were maintained as above.

#### Cellular treatments and transfections

Treatments with FA were performed as stated in the individual protocols and figure legends.

Transfections were performed using Lipofectamine® 3000 (ThermoFisher Scientific) or Fugene® HD transfection reagent (Promega) and expression of the genes was allowed for 15-48 hours; transfection with siRNAs was performed with Lipofectamine RNAiMAX transfection reagent and silencing was allowed for 72 hours.

#### Cell cycle synchronization

HeLa cells were synchronized at G1/S of the cell cycle by double thymidine treatment, as described previously (Harper, 2005).

#### Flow cytometry

Cells were harvested, washed with PBS and subsequently fixed in ice-cold methanol for 15 minutes at 20°C. After washing and rehydration in PBS containing 1% BSA, the cells were stained with 20 μg/ml of propidium iodide diluted in PBS 1% BSA and 10 μg/ml RNase A for 30 minutes at RT. For EdU analysis, cells were incubated with 10 μM EdU before harvesting. EdU was detected with a Click-iT EdU Cell Proliferation Assay kit (ThermoFisher Scientific). Cells were analyzed on a BD FACSCalibur flow cytometer. A minimum of 10,000 events was counted. Data analysis was performed using FlowJo.

#### Western blot

Standard protocols for sodium dodecyl sulfate-polyacrylamide gel electrophoresis (SDS-PAGE) and immuno-blotting were used (Henderson and Wolf, 1992). Nitrocellulose membrane (GE Healthcare) or Polyvinylidene fluoride (PVDF) (Bio-Rad) were used to transfer proteins from polyacrylamide gels depending on the antibody. Acquisition was performed with a Bio-Rad ChemiDoc XRS Plus Analyzer or X-Ray film (Scientific Laboratory Supplies). Quantification of western blot bands was performed on ImageLab software (Bio-Rad) or ImageJ after scanning the film.

#### DPC isolation

DPCs were detected using a modified rapid approach to DNA adduct recovery (RADAR) assay (Kiianitsa and Maizels, 2013). In brief, 1.5 to 2 × 10^6^ cells were lysed in 1-4 mL of DPC lysis buffer, containing 6 M guanidinium isothiocyanate, 10 mM Tris–HCl (pH 6.8), 20 mM EDTA, 4% Triton X-100, 1% N-Lauroylsarcosine Sodium and 1% dithiothreitol. DNA was precipitated by adding an equal volume of 100% ethanol. The DNA pellet was washed three times in wash buffer (20 mM Tris HCl pH 7.5, 50 mM NaCl, 1 mM EDTA, 50% ethanol). DNA was solubilised in 1 mL of 8 mM NaOH. A small aliquot of the recovered DNA was digested with 50 μg/ml proteinase K for 1-3 hours at 55°C. DNA concentration was determined using a Quant-iT PicoGreen dsDNA assay kit (ThermoFisher Scientific) according to manufacturer’s instructions. Normalized amounts of dsDNA (typically 50-100 μg) containing the DPCs were digested with benzonase for 1-2 hours at 37°C. After DNA digestion, proteins were precipitated by standard Trichloroacetic Acid (TCA) protocol (Link and LaBaer, 2011) and resolved by SDS-PAGE gel.

#### DPC detection

Total DPCs were visualized by Flamingo Fluorescent Protein Gel Stain (Bio-Rad) as recommended by the manufacturer after electrophoretic separation on polyacrylamide gels. For slot-blot detection of dsDNA, 100-200 ng of DNA were incubated with proteinase K to digest the crosslinked proteins, diluted in Tris/Borate/EDTA (TBE) buffer and applied to nylon membrane (GE Healthcare). The membrane was blotted with an anti-dsDNA antibody and developed as in “western blot.”

#### Cellular fractionation

HEK293 cells were incubated in 2x volumes of Buffer A (10mM HEPES pH 7.4, 10mM KCl, 340mM sucrose, 10% glycerol, 2mM EDTA, 10mM NEM; protease and phosphatase inhibitors and 0.1% Triton X-100) on ice for 5 minutes. Samples were spun (500 g, 3 minutes, 4°C) and the supernatants (cytosolic fraction) collected and stored. Nuclei were washed twice (500 g, 3 minutes, 4°C) with Buffer A without Triton X-100 and burst in 2x volumes of hypotonic Buffer B (3 mM EDTA, 0.2 mM EGTA, 5 mM HEPES pH 7.9; 10 mM NEM; protease and phosphatase inhibitors) on ice for 10 minutes. After centrifugation at 1,700 g for 3 min, the supernatant (nuclear soluble fraction) was collected and stored. The pellet (chromatin fraction) was washed twice (5,000 g for 5 minutes) with Buffer B. For chromatin nuclease extracts, chromatin fraction was washed in benzonase buffer (25 mM Tris HCl pH 7.9; 25 mM NaCl; 2.5 mM KCl; 3 mM MgCl_2_) and incubated in the same buffer with 200 U/ml benzonase on ice until DNA digestion was complete. Sample was spun at 20,000 g for 5 minutes. The supernatant (chromatin soluble fraction) was quantified and analyzed by SDS-PAGE.

FA treatments were performed with 1 mM for 2 hours. Ubiquitylation (MLN7243, Chemietek) and SUMOylation (ML-792, MedChemExpress) inhibitors were typically added 15 minutes earlier and kept for the duration of FA treatment. For experiments involving SPRTN ΔUBZ, transfection was carried out 15 h before the experiment.

#### *In vitro* cleavage reactions

YFP-Topo-1 was expressed in HEK293 cells for 48h. Cells (80% confluence, 15 cm diameter dish) were treated with DMSO or CPT (1-10 μM) for 30 minutes. Lysis was performed with 50 mM Tris HCl pH 7.4, 150 mM NaCl, 1 mM EDTA, 0.5% Triton X-100, 10 mM NEM with phosphatases and proteases inhibitors. After complete lysis, chromatin was spun down at 1000 g for 5 minutes, and digested in 50 mM Tris HCl pH 7.4, 150 mM NaCl, 2 mM MgCl_2_ with 250 U/ml benzonase (150 μl). Extracts were quantified, normalized, and brought to volume. Chromatin proteins were denatured with 1% SDS for 10 minutes on ice (total volume 200 μl). 1% Triton X-100 was added to the sample, and finally diluted 5X with IP buffer (50 mM Tris HCl, 150 mM NaCl) to 1 ml. YFP-Topo-1 was captured using GFP-trap® beads (Chromotek) for 2 hours at 4°C. Beads were washed 5 times in IP buffer. YFP-Topo-1 was eluted from beads using 50 μL of 200 mM glycine pH 2.5, then quenched with 5 μL Tris pH 10.5. *In vitro* cleavage reactions were typically carried out in 20 μL (Vaz et al., 2016). Eluted YFP-Topo-1 (3-5 μl) was incubated with recombinant SPRTN (2-4 μg) in 25 mM Tris HCl pH 7.4, 150 mM NaCl for 16 hours at 37°C. Reactions were analyzed by western blotting.

#### Co-immunoprecipitations

Cells were transfected with the plasmids of interest using Lipofectamine® 3000 or FuGene. For experiments involving SPRTN ΔUBZ, transfection was carried out 15h before the experiment. Following treatment with FA (1mM for 1h), cells were washed twice with ice-cold PBS, and the cell pellet lysed with 1 mL of ice-cold IP lysis buffer (50 mM Tris-HCl pH 7.4, 150 mM NaCl, 0.5% NP-40, 10 mM NEM, protease and phosphatase inhibitors) containing 250 U/ml of benzonase. After complete digestion, lysates were cleared at 500 g for 10 minutes. Extracts were quantified, normalized, and brought to volume (1 ml). Flag-tag protein complexes were captured using the Anti-FLAG® M2 Affinity Gel or Anti-FLAG® M2 magnetic beads (Sigma-Aldrich) for 4 hours at 4°C. Beads were washed 5 times with IP lysis buffer and eluted using 3X Flag® peptide (Sigma-Aldrich). Flag-tag protein complexes were analyzed by western blotting.

#### Denaturing pull-down of SPRTN

SPRTN-SSH was expressed in HEK293 cells for 24h. Following treatment with FA (1 mM for 1h), cells were washed twice with ice-cold PBS, and cell pellet was lysed with 1 mL of ice-cold IP lysis buffer (50 mM Tris-HCl pH 7.4, 150 mM NaCl, 0.5% NP-40, 10 mM NEM, protease and phosphatase inhibitors) containing 250 U/ml of benzonase. After complete digestion, lysates were cleared at 500 g for 10 minutes. Extracts were quantified, normalized, and brought to volume. Proteins were denatured with 1% SDS at 55°C for 10 minutes (total volume 1 ml). Samples were diluted 10X with 50 mM Tris HCl pH 7.4, 150 mM NaCl, 1% Triton X-100. SPRTN-SSH was captured in a 10 mL volume with Strep-Tactin®Sepharose® resin (IBA lifesciences) for 3h at 4°C. Beads were washes 3 times with 50 mM Tris HCl pH 7.4, 150 mM NaCl, 0.05% Triton X-100, and bound proteins eluted in 50 μL 2X Laemmli buffer.

#### UV laser microirradiation

Cells were seeded onto 10 mm No. 1 glass coverslips (VWR) 24 hours before the experiment. Twenty minutes before UV-A laser, cells on coverslips were treated with 10 μg/mL Hoechst and 10 μM EdU, and micro-irradiated using 355 nm pulsed laser connected to a Nikon TE2000 microscope. After recovery for the indicated times, cells were pre-extracted on ice for 5 minutes with 25 mM HEPES (pH 7.4), 50 mM NaCl, 1 mM EDTA, 3 mM MgCl_2_, 300 mM sucrose, and 0.5% Triton X-100. Cells were fixed in 4% cold formaldehyde PBS for 15 minutes at room temperature, washed in PBS and incubated in blocking solution 5% BSA in PBS overnight at 4°C. S-phase cells were stained with a Click-iT EdU Cell Proliferation Assay kit according to the manufacturer’s instructions (ThermoFisher Scientific). Cells were then incubated with the indicated primary and Alexa Fluor 488 and 568 secondary antibodies in 2.5% BSA PBS solution for 1 hour at room temperature. Cover glasses were washed three times for 5 minutes each in between antibodies and mounted in ProLong Diamond with DAPI (ThermoFisher Scientific). Immunofluorescence images were captured using a Nikon Ni-E epifluorescent microscope under a 60X objective. Data were analyzed using ImageJ. Signal for γH2AX was used as a marker of DNA breaks to outline the laser stripes, and the signal intensity in the channel of interest was then quantified. Nuclear background signal was subtracted from the intensity of the region of interest. Measurements were normalized to the earliest time-point DMSO control.

#### Immunofluorescence

Cells were seeded on glass coverslips 24 hours before experiments. Cells were washed with PBS, fixed for 10 min with ice-cold 3.7% formaldehyde and washed three times in PBS. Cells were fixed for additional 5 min with ice-cold methanol. For pre-extraction, cells were washed once with ice-cold CSK buffer (10 mM Pipes pH 7.0, 100 mM NaCl, 300 mM sucrose, and 3 mM MgCl_2_), and pre-extracted twice for 2 min with ice-cold CSK containing 0.25% Triton X-100. After pre-extraction, cells were washed with ice-cold CSK buffer and fixed as described above. After rehydration in PBS, cells were blocked O/N with PBS containing 5% BSA. Coverslips were incubated for 1 h with primary antibodies in PBS/2.5% BSA and then washed five times with PBS and incubated with appropriate secondary antibodies coupled to Alexa Fluor 488 or 594 fluorophores in PBS/2.5% BSA. DNA was stained 10 μg/ml DAPI in PBS. After washes in PBS, coverslips were dipped in water and mounted on glass slides using ProLong Gold Antifade Mountant (ThermoFisher Scientific). Images of immunostained cells were acquired with an epifluorescent microscope (Nikon Ti-E) and foci analysis was performed using ImageJ automated counting. Representative images and co-localization analysis were acquired using a Zeiss LSM780 confocal microscope system.

#### DNA fiber combing

DNA fiber combing was performed according to GenomicVision instructions. Asynchronous HEK293 cells were labeled with 30 μM CldU (Sigma-Aldrich) for 30 minutes and then with 250 μM IdU (Sigma-Aldrich) for additional 30 minutes. Treatment with 450 μM FA was concomitant with IdU incubation. When specified, 50 μM 2-D08 (SUMOylation inhibitor, Sigma-Aldrich) or 5 μM MLN7243 (ubiquitylation inhibitor, Chemietek) were added 15 minutes before CldU incubation and kept for the entire duration of the experiment; alternatively, DMSO was used. Cells were kept at 37°C for the length of the experiment. DNA replication was inhibited with 1x ice-cold PBS. DNA extraction and combing were performed with a FiberPrep® DNA extraction Kit (Genomic Vision) following manufacturer’s instructions. For fiber staining, rat anti-BrdU (for CldU) (Ab6326, Abcam) and mouse anti-BrdU (for IdU) (347580, BD Biosciences) were used. Anti-rat Cy5 (AB6565, Abcam) and anti-mouse Cy3.5 (Ab6946, Abcam) were the respective secondary antibodies. Coverslips were scanned on a Genomic Vision FiberVision® platform. Quantification of IdU-labeled DNA tract lengths was done with FiberStudio® v0.15 software on at least 170 unidirectional fibers/condition. Resulting “Tract length” values (Kb) were represented in box and whiskers plots showing the median (horizontal band) with the 1^st^ and 3^rd^ quartile range (box) (the dots indicate the outliers) and statistically analyzed using GraphPad Prism (two-tailed Mann-Whitney test).

#### Colony-forming assay

HeLa cells were seeded at low density in 6-well plates and incubated overnight. Cells were exposed to increasing doses of FA diluted in cold DMEM for 20 minutes at 4°C, washed in warm PBS and incubated in fresh warm medium at 37°C for 7-10 days. After fixation and staining (1x PBS, 1% methanol, 1% formaldehyde, 0.05% crystal violet), the number of clones was counted using an automated colony counter GelCount (Oxford Optronix). The number of colonies in treated samples was expressed as a percentage of colony numbers in the untreated samples.

#### Sister chromatid exchange assay

HeLa cells were grown in presence of 15 μM BrdU for 48 hours. After this incubation time, cells were treated for 10 minutes with 450 μM FA and DMSO or 10 μM 2-D08. After 2 washes with 1x PBS, cells were incubated for 16 hours at 37°C with medium containing 15 μM BrdU, 30 ng/ml colcemid solution, DMSO or 10 μM 2-D08. Cells were collected, resuspended in pre-warmed optimal hypotonic solution (Genial Helix) and incubated at 37°C for 20 minutes. Cells were fixed with methanol:acetic acid (3:1, v/v). Staining was performed according to (Clare, 2012).

### Quantification and statistical analysis

All experiments were performed at least two times, with similar results. The statistical method used for comparison between experimental groups was an unpaired t test carried out using GraphPad Prism8. Statistical significance was expressed as a p value, where p < 0.05 was considered a statistically significant difference.

## Data Availability

• All raw western blots and analysis data have been deposited at Mendeley data and are publicly available as of the date of publication. The DOI is listed in the key resources table. • This paper does not report original code. • Any additional information required to reanalyze the data reported in this paper is available from the lead contact upon request.

## Supplemental information

Document S1. Figures S1–S6

Document S2. Article plus supplemental information
