# Supplementary material for: The protease SPRTN and SUMOylation coordinate DNA-protein crosslink repair to prevent genome instability
Source: Cell Rep. 2021 Dec 7;37(10):110080. doi: 10.1016/j.celrep.2021.110080 (PMC8674535; doi:10.1016/j.celrep.2021.110080)
Supplement: Document S1. Figures S1–S6 [file mmc1.pdf]

Cell Reports, Volume *37*

Supplemental information

The protease SPRTN and SUMOylation coordinate DNA-protein crosslink repair to prevent genome instability

Annamaria Ruggiano, Bruno Vaz, Susan Kilgas, Marta Popović, Gonzalo Rodriguez-Berriguete, Abhay N. Singh, Geoff S. Higgins, Anne E. Kiltie, and Kristijan Ramadan

Figure S1

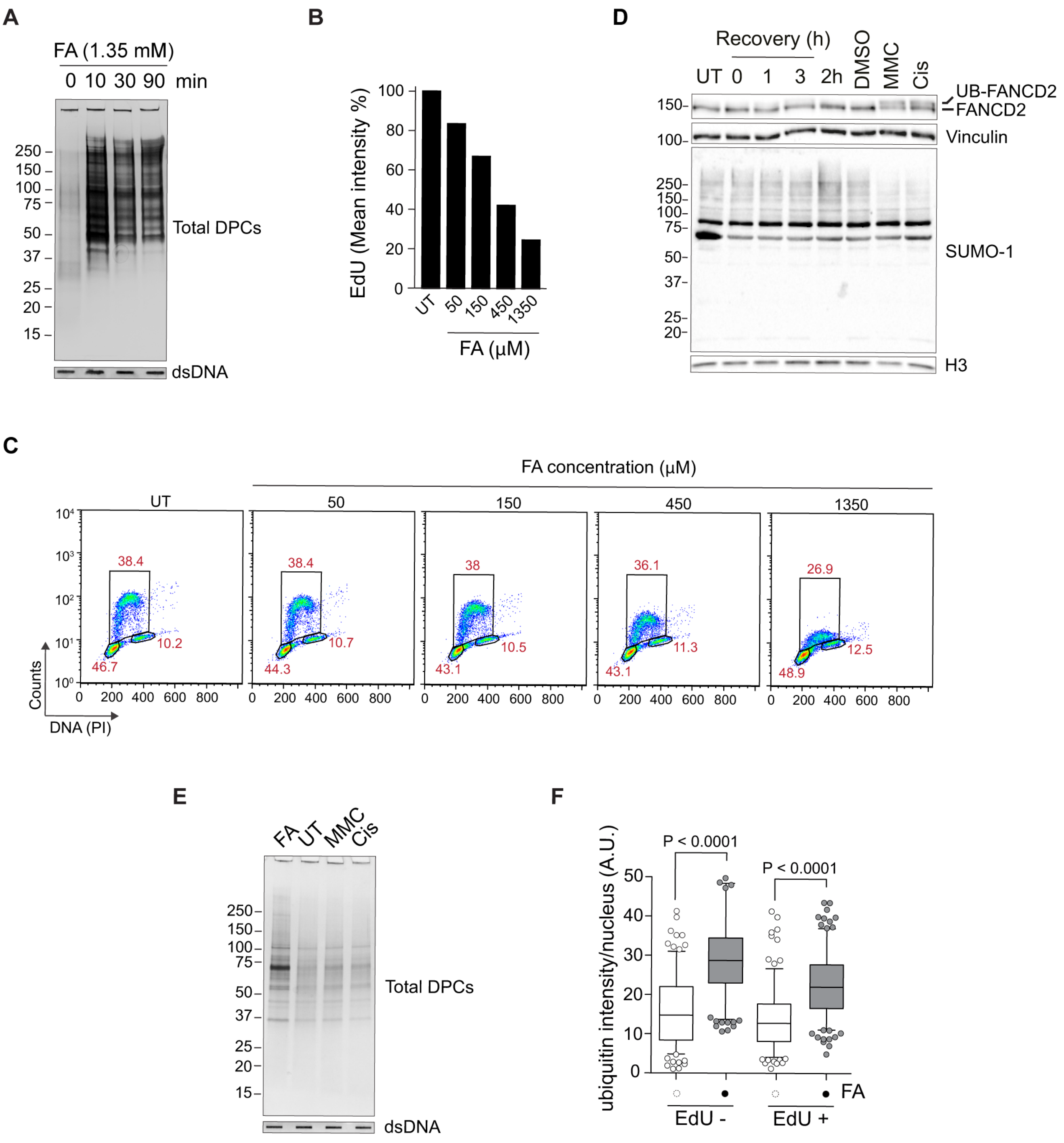

Supplementary figure 1. Formaldehyde treatment causes DPC formation without activating the Fanconi anemia pathway. Related to Figure 1.

(A) HeLa cells were exposed to 1.35 mM FA at 37°C for the indicated times. Total DPCs were isolated by RADAR and analysed by SDS-PAGE followed by Flamingo™ protein gel staining. Slot blot with anti-dsDNA was used as a loading control.

(B) HeLa cells were exposed to increasing concentrations of FA for 30 minutes at 37°C in the presence of EdU (10 μM). EdU incorporation was measured by flow cytometry. Graph represents percentage of EdU mean intensity in replicative cells.

(C) Representative dot-plot graphs of the experiment in (B).

(D) HEK293 cells were treated with 1 mM FA for 20 minutes at 4°C and recovered for the indicated times, or treated continuously for 2 hours. Alternatively, cells were treated with 1 μM Mitomycin C (MMC) or 5 μM cisplatin (Cis) for 24 hours. Total extracts were analysed by western blot for the indicated proteins. Representative of 3 independent experiments.

(E) HeLa cells were exposed to 1 μM Mitomycin C (MMC) or 5 μM cisplatin (Cis) for 2 hours. Total DPCs were isolated by RADAR and analysed by SDS-PAGE followed by Flamingo™ protein gel staining. Slot blot with anti-dsDNA was used as a loading control.

(F) Quantification of the ubiquitin signal intensity using ImageJ (from Figure 1B).

Figure S2

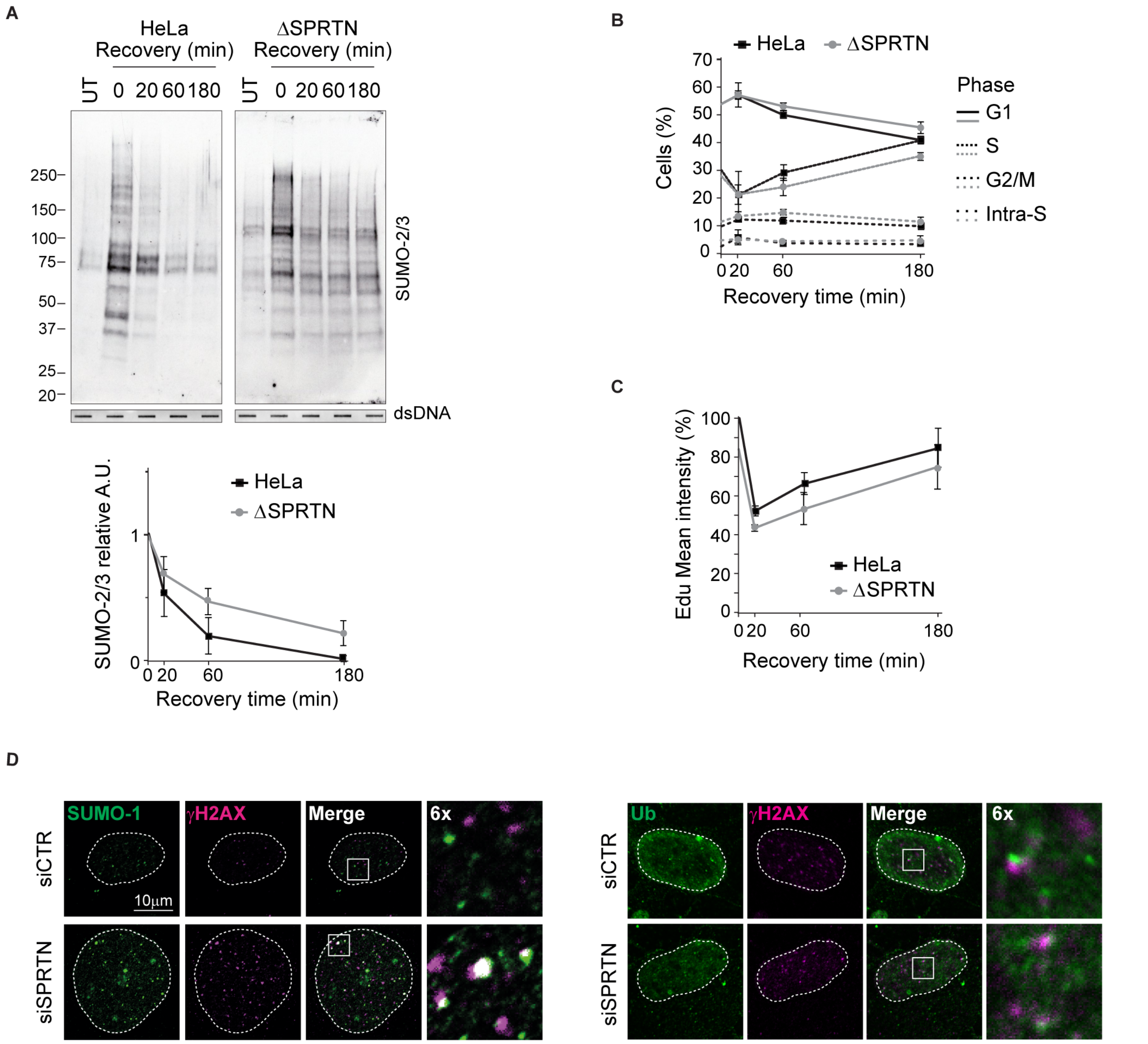

Supplementary figure 2. SUMOylated DPCs accumulate in SPRTN-depleted cells. Related to Figure 3.
(A) Cells were treated and processed as in Figure 3A. Total DPCs were analysed by western blot with anti-SUMO-2/3. Graph shows the mean ± SEM of the relative signal from 3 independent experiments.
(B) Parental and ΔSPRTN HeLa cells were treated as in Figure 3A. Cell cycle distribution was determined by FACS analysis of the DNA content and EdU labeling (n=2, mean ± SD).
(C) Parental and ΔSPRTN HeLa cells were treated as in Figure 3A and EdU mean intensity was determined by FACS analysis (n=2, mean ± SD).
(D) SPRTN was depleted by siRNA in U2OS cells for 3 days. Cells were pre-extracted, fixed and immuno-stained with the indicated antibodies.

Figure S3

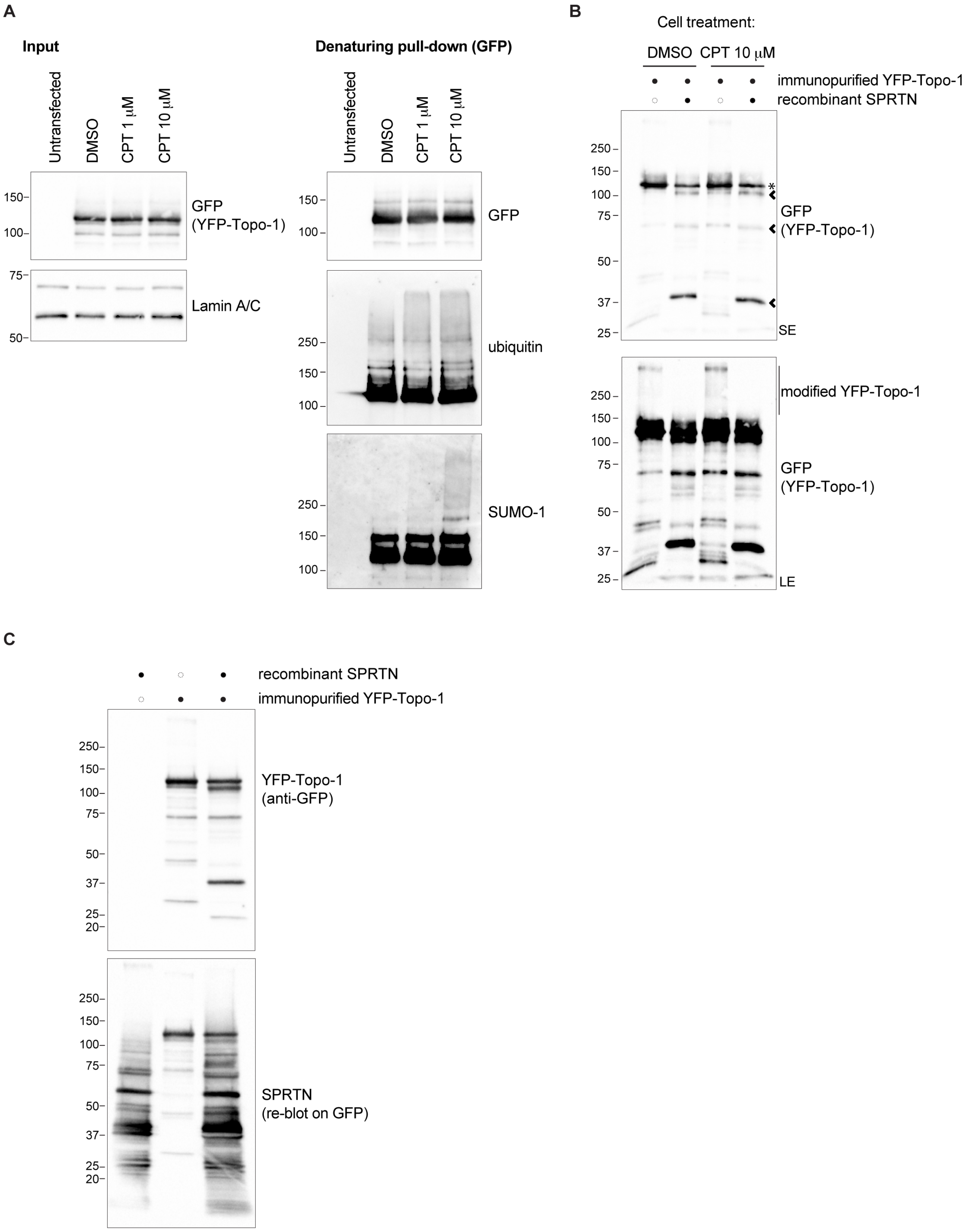

A
Input
Untransfected
DMSO
CPT 1 μM
CPT 10 μM
150
100
GFP
(YFP-Topo-1)
75
50
Lamin A/C
Denaturing pull-down (GFP)
GFP
250
ubiquitin
SUMO-1
B
Cell treatment:
DMSO
CPT 10 μM
immunopurified YFP-Topo-1
recombinant SPRTN
250
150
100
75
50
37
25
*
SE
modified YFP-Topo-1
LE
C
recombinant SPRTN
immunopurified YFP-Topo-1
YFP-Topo-1
(anti-GFP)
20
SPRTN
(re-blot on GFP)

Supplementary figure 3. SUMOylation and ubiquitylation of Topo-1 do not favour SPRTN-dependent proteolysis *in vitro*. Related to Figure 4.

(A) HEK293 cells overexpressing YFP-Topo-1 were treated with DMSO, 1 µM or 10 µM CPT for 1 hour. YFP-Topo-1 was purified from the chromatin fraction under denaturing conditions. The purified protein was analysed by western blot with the indicated antibodies. Representative of 2 independent experiments.

(B) YFP-Topo-1 purified in (A) was incubated with recombinant SPRTN at 37°C for 16 hours. The reactions were analysed by western blot. The asterisk indicates the unmodified, uncleaved Topo-1; the arrowheads mark the major cleavage products. Representative of 3 independent experiments. SE, short exposure; LE, long exposure.

(C) Recombinant SPRTN alone (lane 1) was analysed by western blot with anti-GFP antibody to exclude crossreactivity.

Figure S4

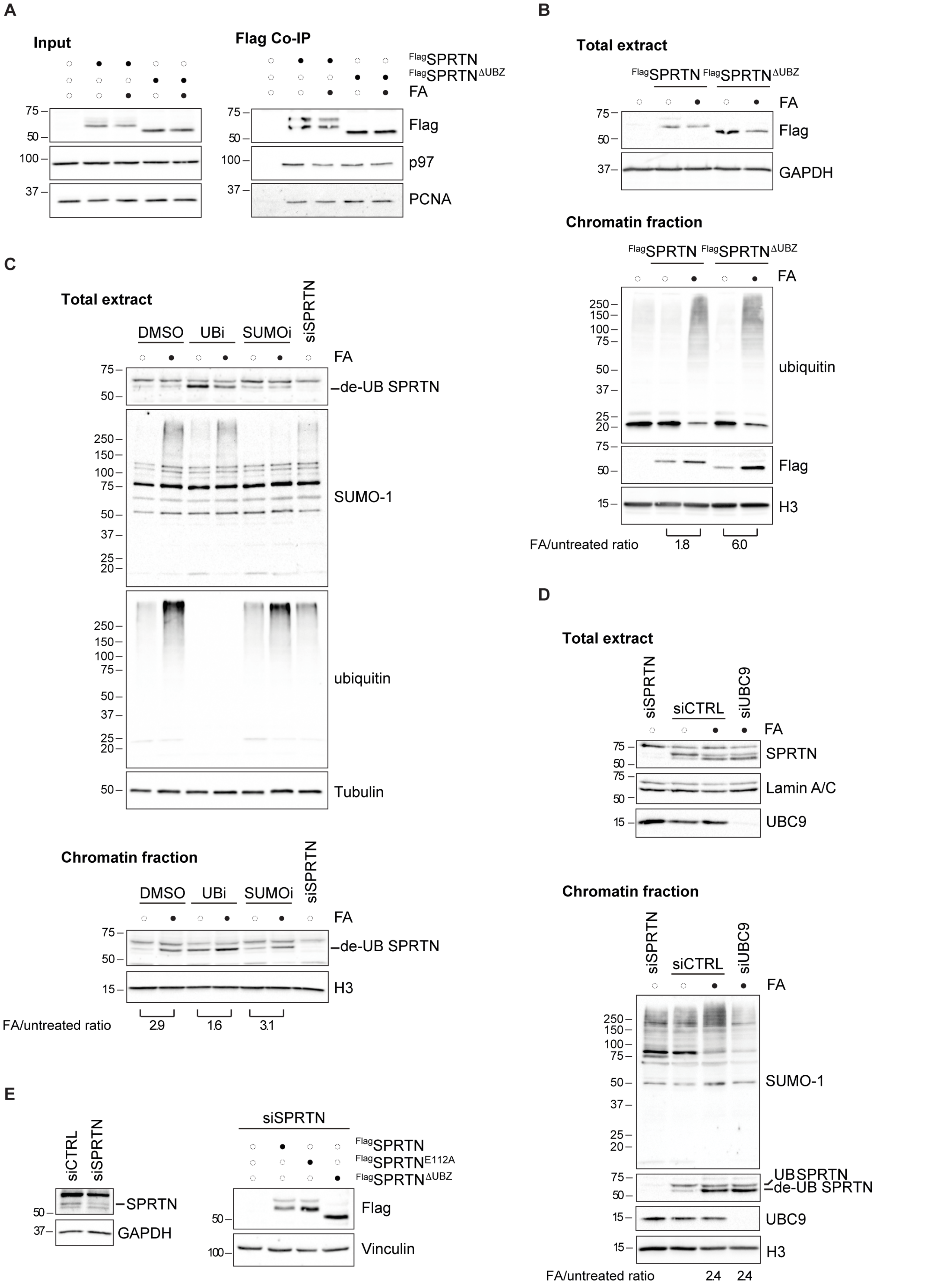

A
Input
Flag Co-IP
FlagSPRTN
FlagSPRTNΔUBZ
FA
75
50
100
37
Flag
p97
PCNA
B
Total extract
FlagSPRTN FlagSPRTNΔUBZ
FA
Flag
GAPDH
Chromatin fraction
FlagSPRTN FlagSPRTNΔUBZ
FA
250
150
100
75
50
37
25
20
15
ubiquitin
Flag
H3
FA/untreated ratio
1.8
6.0
C
Total extract
DMSO
UBi
SUMOi
siSPRTN
FA
de-UB SPRTN
SUMO-1
ubiquitin
Tubulin
Chromatin fraction
DMSO
UBi
SUMOi
siSPRTN
FA
de-UB SPRTN
H3
FA/untreated ratio
2.9
1.6
3.1
D
Total extract
siSPRTN
siCTRL
siUBC9
FA
SPRTN
Lamin A/C
UBC9
Chromatin fraction
siSPRTN
siCTRL
siUBC9
FA
SUMO-1
UB SPRTN
de-UB SPRTN
UBC9
H3
FA/untreated ratio
2.4
2.4
E
siCTRL
siSPRTN
SPRTN
GAPDH
siSPRTN
FlagSPRTN
FlagSPRTNE112A
FlagSPRTNΔUBZ
Flag
Vinculin

Supplementary figure 4. SUMOylation and ubiquitylation are not necessary for SPRTN recruitment to chromatin. Related to Figure 5.

(A) HEK293 cells overexpressing Flag-SPRTN and Flag-SPRTN ΔUBZ were processed as in Figure 5A.

(B) HEK293 cells overexpressing Flag-SPRTN or Flag-SPRTN ΔUBZ for 15 hours were treated, where specified, with 1 mM FA for 2 hours. Chromatin was isolated and recruitment of Flag-SPRTN was analysed by western blot. Ratio of SPRTN levels in the FA-treated versus FA-untreated sample is reported. Representative of 2 independent experiments.

(C) HEK293 cells were treated, where specified, with 1 mM FA for 2 hours in presence of DMSO, the ubiquitylation inhibitor MLN7243 (UBi) (5 μM) or the SUMOylation inhibitor ML792 (SUMOi) (1 μM). Chromatin was isolated and recruitment of endogenous SPRTN was analysed by western blot. Ratio of SPRTN levels in the FA-treated versus FA-untreated sample is reported for each pair. Representative of 3 independent replicates.

(D) siControl- (siCTRL) or siUBC9-silenced HEK293 cells were treated, where specified, with 1 mM FA for 2 hours. Chromatin was isolated and recruitment of endogenous SPRTN was analysed by western blot. Ratio of SPRTN levels in the FA-treated versus FA-untreated sample is reported. Representative of at least 3 independent replicates.

(E) Control cell extracts for experiment in Figure 5D. Western blots confirm SPRTN depletion and overexpression of the specified variants.

Figure S5

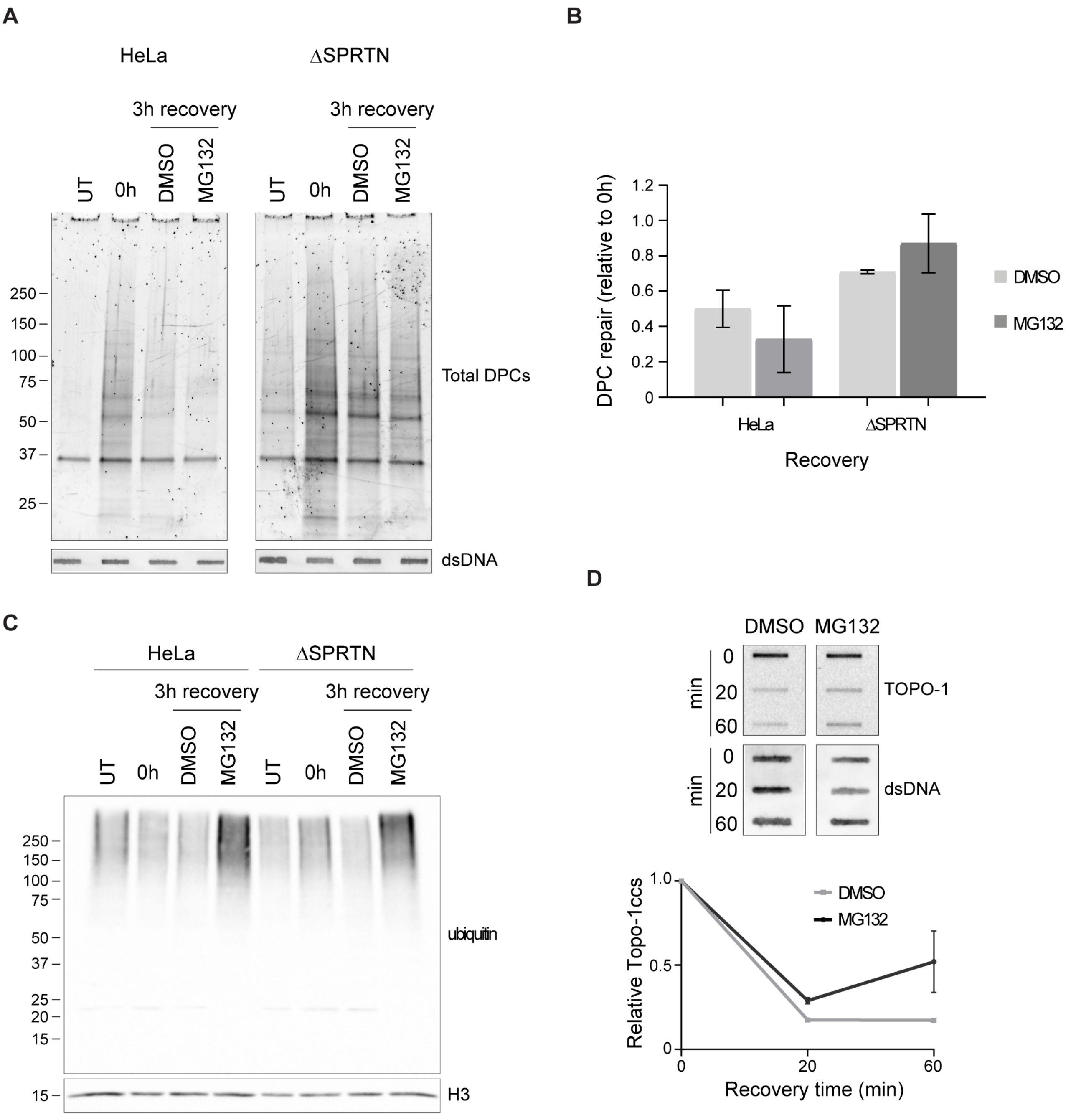

Supplementary figure 5. Effect of proteasome inhibition on DPC repair. Related to Figure 5.

(A) Parental or ΔSPRTN HeLa cells were treated with 1.35 mM FA for 10 minutes at 37°C and allowed to recover for 3 hours in presence of DMSO or 10 µM MG132. Total DPCs were isolated by RADAR and detected with Flamingo ™ protein gel staining.

(B) Quantification of 2 independent experiments performed as described in (A). The amount of total DPCs at the 3-hour time-point compared to 0 hour (marking the end of FA treatment) is reported.

(C) Total extracts of cells from experiment in (A) were analysed by western blot to confirm the effect of MG132 on cellular ubiquitin levels.

(D) HeLa cells were treated with low doses of CPT (50 nM) for 1 hour and recovered for the indicated times in the presence of 10µM MG132. DPCs were isolated by RADAR and analysed by slot blot with antibodies against Topo-1. Slot blot with anti-dsDNA was used as loading control (n = 2, mean ± SD).

Figure S6

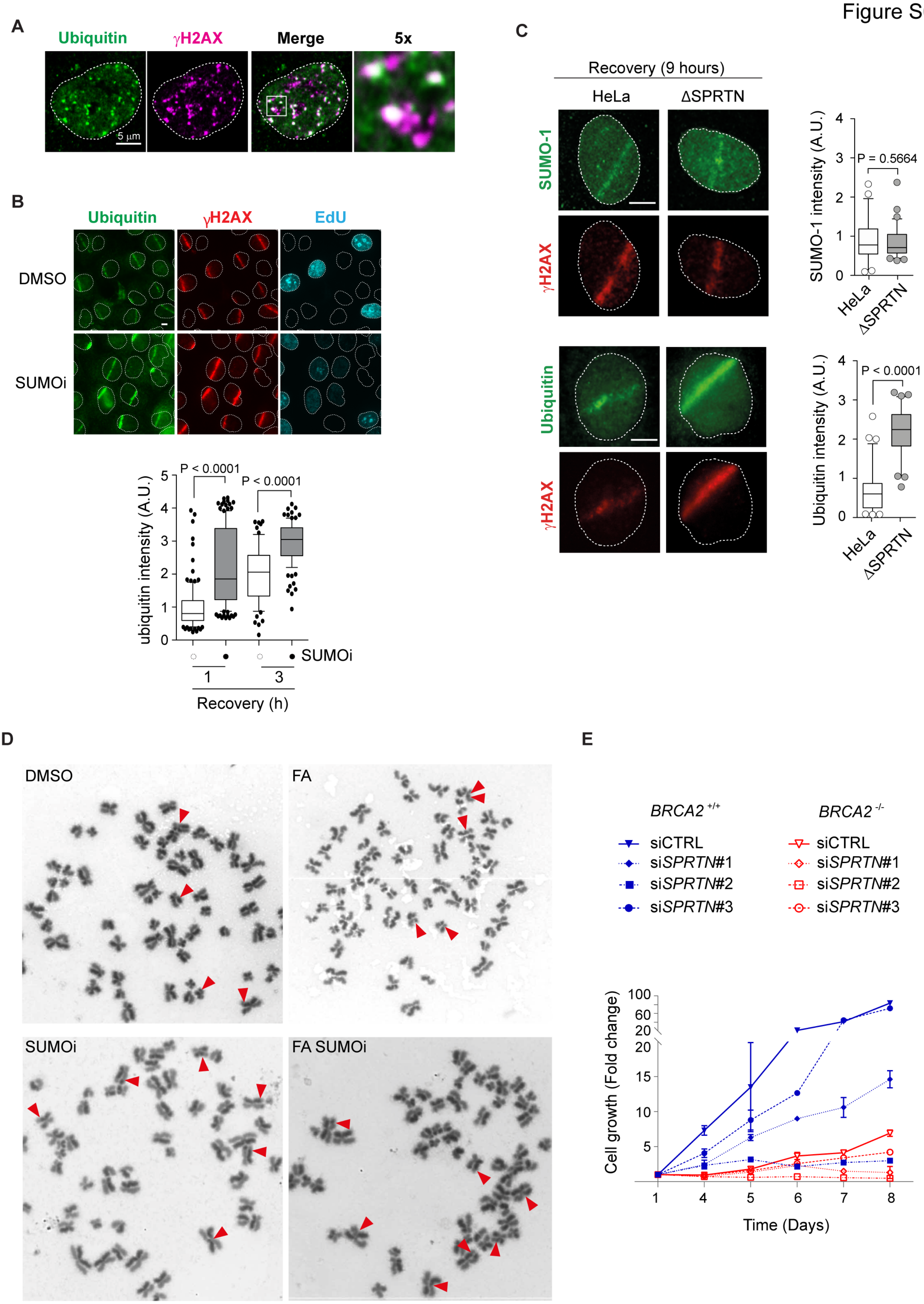
A
Ubiquitin
γH2AX
Merge
5x
5 µm
B
Ubiquitin
γH2AX
EdU
DMSO
SUMOi
ubiquitin intensity (A.U.)
P < 0.0001
P < 0.0001
SUMOi
1
3
Recovery (h)
C
Recovery (9 hours)
HeLa
ΔSPRTN
SUMO-1
γH2AX
Ubiquitin
γH2AX
SUMO-1 intensity (A.U.)
P = 0.5664
HeLa
ΔSPRTN
Ubiquitin intensity (A.U.)
P < 0.0001
HeLa
ΔSPRTN
D
DMSO
FA
SUMOi
FA SUMOi
E
BRCA2 +/+
BRCA2 -/-
siCTRL
siSPRTN#1
siSPRTN#2
siSPRTN#3
siCTRL
siSPRTN#1
siSPRTN#2
siSPRTN#3
Cell growth (Fold change)
Time (Days)

Supplementary figure 6. SUMO suppresses homologous recombination at DPC-induced DNA damage sites. Related to Figure 6.

(A) Confocal microscopy of RPE-1 cells treated as described in Figure 6A. Three hours after treatment cells were fixed and immunostained with the indicated antibodies.

(B) HeLa cells were sensitized with Hoechst 33258 for 30 minutes prior to laser microirradiation. EdU was added during the sensitisation time. Following DNA damage, cells were allowed to recover in the presence of DMSO or 25 μM 2-D08 (SUMOi) for the indicated times. Cells were then pre-extracted, fixed and immunostained with the indicated antibodies. Signal at DNA damage sites was quantified using ImageJ and statistical significance calculated using unpaired t-test (100-140 stripes). Scale bar: 5 μM.

(C) UV laser microirradiation was performed in parental HeLa and ΔSPRTN cells as described in (B). Cells were allowed to recover from damage for the indicated times. Cells were then pre-extracted, fixed and immunostained with the indicated antibodies. Signal at DNA damage sites was quantified in EdU positive cells (100 stripes) using ImageJ and statistical significance calculated using unpaired t-test. Scale bar: 5 μM.

(D) Representative metaphases from experiment in Figure 6E.

(E) SPRTN was depleted at day 2 and day 5 by three siRNA sequences in either BRCA2-deficient (DLD1-/-) or complemented (DLD1+/+) cells. Cells were counted manually for the indicated time using trypan blue to exclude dead cells (n=2, mean ± SD).
